# Epidemiological patterns of chronic kidney disease attributed to type 2 diabetes from 1990-2019

**DOI:** 10.3389/fendo.2024.1383777

**Published:** 2024-04-17

**Authors:** Xiaoxiao Ding, Xiang Li, Yun Ye, Jing Jiang, Mengsang Lu, Lv Shao

**Affiliations:** ^1^ Department of Clinical Pharmacy, Beilun District People’s Hospital, Ningbo, China; ^2^ Department of Clinical Laboratory, The Second Hospital of Anhui University of Traditional Chinese Medicine, Hefei, Anhui, China; ^3^ Department of Clinical Pharmacy, Yuyao People’s Hospital, Ningbo, Zhejiang, China

**Keywords:** type 2 diabetes, chronic kidney disease, EAPC, socio-demographic index, epidemiology

## Abstract

**Background:**

This study investigates the burden of chronic kidney disease attributed to type 2 diabetes (CKD-T2D) across different geographical locations and time periods from 1990 to 2019. A total of 204 countries and regions are included in the analysis, with consideration given to their socio-demographic indexes (SDI). The aim is to examine both spatial and temporal variations in CKD-T2D burden.

**Methods:**

This research utilized data from the 2019 Global Burden of Diseases Study to evaluate the age-standardized incidence rates (ASIR), Disability-Adjusted Life Years (DALYs), and Estimated Annual Percentage Change (EAPC) associated with CKD-T2D.

**Results:**

Since 1990, there has been a noticeable increase of CKD age-standardized rates due to T2D, with an EAPCs of 0.65 (95% confidence interval [CI]: 0.63 to 0.66) for ASIR and an EAPC of 0.92 (95% CI: 0.8 to 1.05) for age-standardized DALYs rate. Among these regions, Andean Latin America showed a significant increase in CKD-T2D incidence [EAPC: 2.23 (95% CI: 2.11 to 2.34) and North America showed a significant increase in CKD-T2D DALYs [EAPC: 2.73 (95% CI: 2.39 to 3.07)]. The burden was higher in male and increased across all age groups, peaking at 60-79 years. Furthermore, there was a clear correlation between SDI and age-standardized rates, with regions categorized as middle SDI and High SDI experiencing a significant rise in burden.

**Conclusion:**

The global burden of CKD-T2D has significantly risen since 1990, especially among males aged 60-79 years and in regions with middle SDI. It is imperative to implement strategic interventions to effectively address this escalating health challenge.

## 1 Introduction

Chronic kidney disease (CKD)-Type 2 Diabetes (T2D) is a common chronic complication resulting from diabetes mellitus, characterized by alternating or sustained albuminuria and/or a progressive decline in glomerular filtration rate. In the absence of comprehensive treatment approaches, CKD-T2D frequently progresses to End-Stage Renal Disease (ESRD) (1). Importantly, older adults with T2D, especially those who have had the disease for ten years or more, have a higher likelihood of developing CKD compared to non-diabetic individuals (2). With an aging population, increased life expectancy, and changing lifestyle patterns, the CKD-T2D is rising, contributing significantly to the global increase in ESRD (3).

Research has indicated that obesity, hypertension, and being male are key risk factors for the onset and advancement of CKD-T2D (4, 5). Furthermore, the occurrence and fatality rates of CKD resulting from diabetes are closely linked to socio-economic, cultural, and healthcare management factors at the national level, as well as the age of the patient (3, 6). Several studies have examined the global and regional patterns of CKD, including its incidence, incidence, and mortality, and have noted variations based on sex and age groups (7). For instance, Pan and Liu et al. conducted a study on the incidence of diabetes and CKD in China (8), while another study focused on mortality and trends in diabetes cases among individuals under the age of 25 (9). However, there is still a lack of research on the epidemiological aspects of CKD-T2D on a global and regional scale, across all age groups.

Given the lengthy evolution of diabetes mellitus into CKD-T2D and the need for long-term interventions to effectively manage CKD-T2D, it is imperative to prioritize early prevention, timely detection, and prompt intervention. In light of the consequences of CKD associated with T2D, our study utilizes age-standardized rates (ASRs) to quantify its incidence and disability trends. These include the age-standardized incidence rate (ASIR) and the age-standardized disability-adjusted life year (DALY) rate. Understanding the global impact of CKD-T2D across all age groups is crucial for devising strategies to prevent and ultimately reduce its incidence.

This study aims to analyze the global burden of CKD-T2D in individuals across all age groups. The data from the Global Burden of Disease (GBD2019) study is examined to accomplish this objective. The analysis encompasses the observation of disease trends over the period from 1990 to 2019, the identification of disparities between different countries and regions, and the evaluation of variations by age.

## 2 Method

### 2.1 Data source and measures of burden

The Global Burden of Disease (GBD) 2019 is a comprehensive international initiative that provides estimates on the impact of 369 diseases and injuries across 204 countries and territories from 1990 to 2019 (10). To gather data for each specific disease or injury, the GBD utilizes a wide range of sources including 7,333 national and 24,657 sub-national vital registration systems, 16,984 scholarly publications, and 1,654 household surveys, as well as other relevant sources such as population censuses, healthcare usage records, and satellite imagery (10). This study is updated annually to incorporate refinements to the range of diseases, data sources, and methodologies. These updates aim to accurately capture yearly variations in the same diseases and injuries, stratified by age, sex, country, and region, using standard epidemiological and health metrics such as incidence, prevalence, mortality rates, and DALYs (10).

DALYs, a crucial metric in epidemiological research, quantify the overall burden of disease by measuring the total years of healthy life lost from disease onset to death. This measurement encompasses both years lost due to premature mortality and years lost due to disability, and can be expressed as either a numerical count or a rate (11). This study is based on a publicly available database and does not require ethical approval.

### 2.2 Clinical criteria

This study focuses on CKD-T2D, which is classified under ICD-10 codes E11.2 to E11.29 and under ICD-9 codes 250.40 and 250.42. According to the 2019 Global Burden of Disease Study guidelines, diabetes is defined as a fasting blood glucose level equal to or greater than 126 mg/dL (7 mmol/L) or through reported diabetes treatment. CKD-T2D, a subtype of chronic kidney disease caused by Type 2 Diabetes, is characterized by a duration exceeding three months. It is primarily identified through a urinary albumin/creatinine ratio exceeding 30 mg/g and/or an estimated glomerular filtration rate below 60 mL/min per 1.73 m² (12).

### 2.3 Estimation of attributable risk factors for CKD-T2D

The GBD 2019 study estimated the disease burden attributable to 87 risk (or risk cluster) factors at the global, regional, and national levels. Population Attributable Fractions (PAF) are employed to assess the contribution of specific risk factors to disease or mortality within an entire population, as well as the proportion by which disease incidence or mortality can be reduced if the population were at the theoretical minimum risk exposure level. The product of PAF with the disease’s DALYs and deaths represents the DALYs and deaths attributed to that risk factor. The attributed standardized rates were used to measure the attributable disease burden of global CKD risk factors.


$$PAF_{asly}=\sum_{x=1}^{k}RR_{asy}(x)P_{asly}(x)-\frac{1}{\sum_{x=1}^{k}RR_{as}(x)P_{asly}(x)}$$

where a is the age group, s is the sex, l is the location, and y is the year; PAFasly is the PAF for the burden of diseases due to T2D; RR is the relative risks between exposure level x (from 1 to k) of T2D and the burden of CKD; and P is the proportion of the population exposed to T2D.

### 2.4 Statistical analysis

The Global Burden of Disease studies examine the impact of 329 diseases across 204 countries and territories. These countries are divided into 21 clusters based on epidemiological and geographical factors (13). For our research, we used the Socio-Demographic Index (SDI) to categorize countries. The SDI combines various factors such as per capita income, educational attainment, and fertility rates among women under 25 years old. The index ranges from 0, representing lower income, education, and higher fertility rates, to 1, indicating higher income, education, and lower fertility rates (14). Based on the SDI, countries were classified into five tiers: low (below 0.46), low-middle (0.46–0.60), middle (0.61–0.69), high-middle (0.70–0.81), and high (above 0.81).

Using global standard population data from the GBD 2019 study, Age-Standardized Rates (ASRs) for incidence and DALY (per 100,000 population), along with their 95% Uncertainty Intervals (UIs), were calculated using the direct standardization method. To examine the temporal trends in CKD-T2D incidence and DALY rates from 1990 to 2019 globally, Estimated Annual Percent Changes (EAPCs) and their 95% Confidence Intervals (CIs) were computed. EAPC is a commonly used measure to assess rate trends over specified time periods. It was determined by fitting a regression line to the natural logarithm of the rates (y = α + βx + ϵ), where y represents the natural logarithm of the rate and x represents the calendar year. The EAPC calculation involved multiplying 100 by (exp[β]−1), and its 95% UI was obtained using linear regression modeling (15). All statistical analyses were performed using R software (version 4.2.1), and a P-value less than 0.05 was considered statistically significant.

## 3 Result

### 3.1 Global trends and burden of CKD-T2D

**Figure 1:**
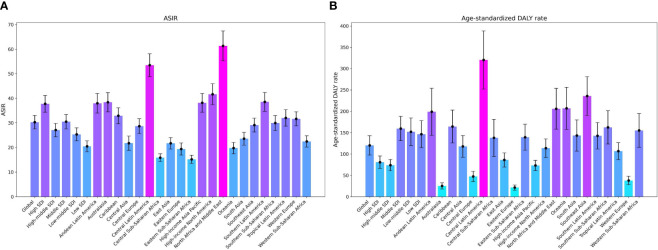
The ASRs of Global Burden of Chronic kidney disease attributed to type 2 diabetes mellitus by region: age-standardized DALY rate and ASIR in 2019. **(A)** ASIR, **(B)** Age-Standardized DALY Rate; ASRs, age-standardized rates; ASIR, age-standardized Incidence rate; DALY, disability adjusted life-year.

**Figure 2:**
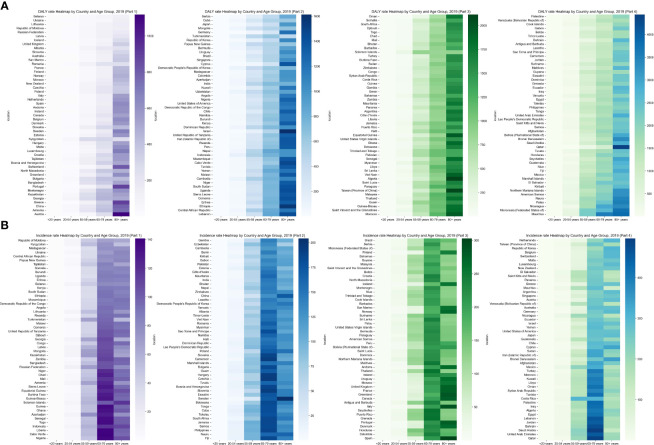
Global Heatmap of Chronic kidney disease attributed to type 2 diabetes mellitus 2019 by Country and Age Group: **(A)** Age-Standardized DALY Rate, **(B)** ASIR. DALY, Disability-Adjusted Life-Year; ASIR, Age-Standardized Incidence Rate.

**Figure 3:**
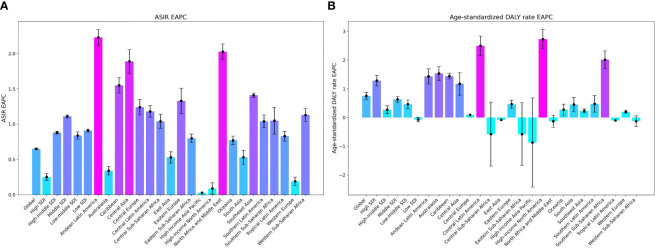
Trends in EAPCs of Chronic kidney disease attributed to type 2 diabetes mellitus by region: age-standardized DALY Rate and ASIR, 1990-2019. **(A)** ASIR, **(B)** Age-Standardized DALY Rate. EAPCs, Estimated Annual Percentage Changes; DALY, disability-adjusted life-year.

The incidence of CKD-T2D was 2,501,248 thousand cases, corresponding to an age-standardized incidence rates (ASIR) of 30.29 per 100,000 population, representing a 21.8% increase since 1990. The number of DALYs attributed to CKD-T2D was 9,870.4 thousand, with an age-standardized rate of 120.2 per 100,000 population, demonstrating an 18.2% increase since 1990 (**Figure 1**, **Tables 1**, **2**). The mortality rate of CKD-T2D showed an upward trend with age, reaching its peak in the 60-79 age group and declining thereafter (**Figure 2B**). The highest incidence rate was observed in the same age group (**Figure 2B**). From 1990 to 2019, both the global age-standardized DALY rate and ASIR of CKD-T2D experienced a slight increase, with EAPCs of 0.65 (95%CI: 0.63 to 0.66) for the ASIR and 0.75 (95%CI: 0.63 to 0.87) for the age-standardized DALY rate, respectively (**Tables 1**, **2**, **Figures 1**, **3**).

**Table 1:** Incidence of chronic kidney disease attributed to type 2 diabetes mellitus in 1990 and 2019 for both sexes and all regions.

location	Numbers_1990	ASR_1990	Numbers_2019	ASR_2019	EAPC_CI	Numbers change
Global	975171(881612-1077106)	24.88 (22.6-27.39)	2501248 (2279950-2740780)	30.29 (27.65-33.05)	0.65 (0.63-0.66)	1.56 (1.52-1.62)
High SDI	359063(324687-397198)	33.61 (30.48-36.95)	710936 (648656-775186)	37.74 (34.43-41.16)	0.25 (0.2-0.3)	0.98 (0.93-1.04)
High-middle SDI	228812(205594-253754)	21.41 (19.36-23.65)	558152 (506331-613450)	27.07 (24.6-29.78)	0.88 (0.86-0.9)	1.44 (1.37-1.5)
Middle SDI	232053(207352-259869)	22.36 (20.07-24.89)	775961 (705254-850164)	30.49 (27.76-33.43)	1.11 (1.09-1.13)	2.23 (2.13-2.32)
Low-middle SDI	117552(105344-131111)	19.17 (17.32-21.27)	349773 (315707-387048)	25.34 (22.95-27.98)	0.84 (0.78-0.89)	1.96 (1.88-2.04)
Low SDI	37221(33397-41594)	15.65 (14.2-17.3)	104990 (94631-116523)	20.48 (18.48-22.69)	0.91 (0.88-0.93)	1.7 (1.64-1.77)
Andean Latin America	4037(3646-4515)	19.96 (18.06-22.26)	21137 (19048-23334)	37.98 (34.1-41.96)	2.23 (2.11-2.34)	4.24 (3.95-4.52)
Australasia	8345(7739-9041)	33.96 (31.6-36.64)	19491 (17621-21480)	38.4 (34.75-42.31)	0.34 (0.28-0.4)	1.34 (1.21-1.48)
Caribbean	5453(4891-6080)	20.72 (18.6-23.15)	17092 (15542-18810)	32.91 (29.94-36.13)	1.55 (1.44-1.66)	2.13 (2-2.26)
Central Asia	6316(5481-7284)	13.01 (11.43-14.88)	17522 (15269-20084)	21.75 (19.19-24.65)	1.89 (1.72-2.06)	1.77 (1.67-1.88)
Central Europe	27966(24834-31685)	18.61 (16.6-20.87)	61605 (55095-68623)	28.65 (25.85-31.68)	1.24 (1.13-1.35)	1.2 (1.1-1.31)
Central Latin America	31707(28362-35621)	36.83 (33.01-41.52)	128815 (117945-139956)	53.46 (49.17-58.15)	1.18 (1.1-1.26)	3.06 (2.83-3.33)
Central Sub-Saharan Africa	2643(2348-2980)	11.78 (10.63-12.97)	8130 (7271-9093)	15.86 (14.22-17.42)	1.04 (0.95-1.14)	2.08 (1.95-2.21)
East Asia	172075(152472-193061)	19.76 (17.64-22.04)	458410 (413635-506945)	21.7 (19.63-24.02)	0.53 (0.45-0.61)	1.66 (1.56-1.76)
Eastern Europe	37727(33334-42629)	13.54 (12.05-15.21)	65215 (57772-73961)	19.38 (17.33-21.82)	1.33 (1.14-1.51)	0.73 (0.68-0.78)
Eastern Sub-Saharan Africa	8779(7887-9759)	12.01 (10.9-13.23)	23720 (21225-26367)	15.19 (13.64-16.83)	0.8 (0.73-0.86)	1.7 (1.65-1.76)
High-income Asia Pacific	73771(67089-81638)	36.22 (33-39.95)	169230 (153066-185899)	38.16 (34.72-41.98)	0.03 (-0.01-0.08)	1.29 (1.19-1.4)
High-income North America	139294(123770-155141)	38.8 (34.84-43.15)	262824 (236671-291204)	41.61 (37.62-45.89)	0.09 (0-0.17)	0.89 (0.82-0.96)
North Africa and Middle East	60320(54043-67275)	35.05 (31.66-38.83)	265941 (241657-294375)	61.33 (55.98-67.44)	2.03 (1.92-2.14)	3.41 (3.28-3.55)
Oceania	476(416-534)	15.17 (13.44-16.89)	1435 (1261-1613)	19.77 (17.47-22.07)	0.77 (0.71-0.83)	2.02 (1.91-2.14)
South Asia	113505(101001-127355)	19.13 (17.16-21.38)	336491 (300753-375009)	23.58 (21.1-26.18)	0.53 (0.44-0.63)	1.96 (1.86-2.08)
Southeast Asia	49853(44639-55492)	19.14 (17.27-21.2)	181865 (164123-200650)	29.11 (26.35-31.92)	1.41 (1.39-1.44)	2.65 (2.54-2.76)
Southern Latin America	13172(11700-14636)	28.35 (25.28-31.4)	32552 (29532-35833)	38.54 (34.99-42.37)	1.04 (0.95-1.13)	1.47 (1.3-1.66)
Southern Sub-Saharan Africa	5893(5253-6661)	21.47 (19.23-24.08)	16918 (15298-18832)	29.93 (27.01-32.99)	1.05 (0.86-1.24)	1.87 (1.79-1.96)
Tropical Latin America	23008(20671-25520)	24.63 (22.18-27.37)	78314 (71206-86595)	32.01 (29.15-35.36)	0.83 (0.77-0.9)	2.4 (2.26-2.58)
Western Europe	176265(158422-195239)	29.12 (26.21-31.99)	293228 (266225-320303)	31.65 (28.77-34.47)	0.19 (0.13-0.25)	0.66 (0.6-0.73)
Western Sub-Saharan Africa	14568(13136-16092)	16.69 (15.1-18.31)	41312 (37220-45693)	22.47 (20.24-24.77)	1.13 (1.04-1.22)	1.84 (1.79-1.89)

**Table 2:** DALYs of chronic kidney disease attributed to type 2 diabetes mellitus in 1990 and 2019 for both sexes and all regions.

location	Numbers_1990	ASR_1990	Numbers_2019	ASR_2019	EAPC_CI	Numbers_ change
Global	4083275 (3296982-4859145)	101.71 (82.95-120.08)	9870472 (8114784-11736440)	120.2 (99.16-142.85)	0.75 (0.63-0.87)	1.42 (1.21-1.61)
High SDI	607346 (503484-719076)	58.49 (48.34-68.99)	1524995 (1252922-1803414)	80.85 (66.99-95.28)	1.28 (1.09-1.46)	1.51 (1.37-1.67)
High-middle SDI	773611 (619606-915550)	72.84 (59.13-86.62)	1495234 (1229298-1766937)	73.88 (61.08-87.11)	0.27 (0.12-0.41)	0.93 (0.74-1.12)
Middle SDI	1512980 (1213592-1800802)	143.21 (117.35-167.24)	3984287 (3268665-4743617)	159.63 (132.14-188.19)	0.63 (0.53-0.73)	1.63 (1.35-1.9)
Low-middle SDI	835984 (648632-1026231)	135.5 (106.29-165.68)	2104038 (1648646-2571907)	152.04 (119.82-184.33)	0.46 (0.3-0.61)	1.52 (1.25-1.8)
Low SDI	350598 (268467-435285)	149.58 (115.74-182.93)	754936 (587975-928133)	146.36 (115.93-178)	-0.07 (-0.14–0.01)	1.15 (0.92-1.39)
Andean Latin America	28307 (21976-34678)	138.91 (108.25-169.77)	110229 (84081-140907)	198.82 (151.5-253.93)	1.43 (1.17-1.69)	2.89 (2.22-3.69)
Australasia	4006 (3197-5016)	17.34 (13.81-21.58)	12751 (9621-16431)	25.2 (19.08-32.44)	1.53 (1.28-1.77)	2.18 (1.71-2.74)
Caribbean	31686 (25281-38216)	121.07 (97.08-146.39)	85229 (66851-105504)	164.33 (129.45-202.86)	1.44 (1.33-1.54)	1.69 (1.34-2.1)
Central Asia	39000 (30089-48789)	78.61 (60.41-98.13)	92942 (72417-114089)	117.66 (93.25-142.98)	1.17 (0.79-1.56)	1.38 (1.03-1.7)
Central Europe	72430 (54818-90303)	48.91 (37.72-60.54)	100975 (76782-127360)	47.48 (36.46-59.44)	0.09 (-0.06-0.24)	0.39 (0.22-0.59)
Central Latin America	138581 (112853-163253)	164.34 (135.96-192)	763212 (606403-925515)	320.2 (255.6-388.56)	2.5 (2.16-2.84)	4.51 (3.9-5.17)
Central Sub-Saharan Africa	34971 (25588-45310)	158.7 (119.27-200.54)	70905 (49798-94949)	137.93 (98.29-181.44)	-0.58 (-0.63–0.53)	1.03 (0.61-1.55)
East Asia	904818 (712046-1092641)	100.28 (80.75-119.3)	1760706 (1419106-2118732)	86.06 (69.83-102.66)	-0.08 (-0.23-0.07)	0.95 (0.63-1.32)
Eastern Europe	50435 (38262-64666)	18.28 (13.99-23.18)	73481 (55967-94257)	21.53 (16.5-27.48)	0.46 (0.33-0.6)	0.46 (0.35-0.58)
Eastern Sub-Saharan Africa	117799 (90317-145993)	160.33 (123.68-199.27)	213357 (167740-261059)	139.28 (110.65-169.71)	-0.58 (-0.65–0.51)	0.81 (0.6-1.05)
High-income Asia Pacific	194215 (164371-222917)	98.87 (83.9-113.3)	339782 (282435-396587)	73.24 (61.75-85.24)	-0.87 (-1.05–0.68)	0.75 (0.58-0.92)
High-income North America	187257 (145444-230870)	53.76 (41.87-66.08)	706432 (566958-843057)	113.61 (91.76-135.29)	2.73 (2.39-3.07)	2.77 (2.43-3.15)
North Africa and Middle East	370212 (293306-453591)	222.15 (175.54-274.43)	863703 (678142-1069675)	205.94 (162.38-253.6)	-0.13 (-0.18–0.08)	1.33 (0.91-1.76)
Oceania	6114 (4687-7626)	182.83 (144.01-224.89)	16119 (12344-20440)	206.91 (164.32-256.32)	0.27 (0.08-0.46)	1.64 (1.17-2.2)
South Asia	734254 (546152-940746)	127.52 (95.75-161.41)	2053209 (1534680-2589368)	143.36 (108.63-179.93)	0.45 (0.2-0.7)	1.8 (1.39-2.32)
Southeast Asia	625786 (499377-750458)	226.44 (184.91-267.62)	1475059 (1195581-1779953)	235.7 (194.17-281.24)	0.23 (0.17-0.3)	1.36 (1.03-1.68)
Southern Latin America	57056 (45283-68254)	124.21 (99.31-148.26)	119596 (94695-145445)	142.69 (113.09-173.69)	0.47 (0.19-0.76)	1.1 (0.91-1.3)
Southern Sub-Saharan Africa	29623 (22020-38253)	106.17 (79.5-135.34)	89625 (68707-111864)	162.32 (125.88-201.31)	2.01 (1.71-2.32)	2.03 (1.67-2.37)
Tropical Latin America	98492 (79198-117442)	106.48 (86.54-126.13)	258295 (210290-307040)	106.63 (87.46-126.49)	-0.1 (-0.22-0.02)	1.62 (1.43-1.82)
Western Europe	222097 (171716-278097)	37.65 (29.23-46.55)	380268 (294052-489271)	37.99 (29.94-47.8)	0.2 (0.15-0.25)	0.71 (0.54-0.9)
Western Sub-Saharan Africa	136135 (99132-173948)	159.76 (118.81-202.51)	284596 (216363-359541)	155.3 (119.85-194.88)	-0.13 (-0.2–0.06)	1.09 (0.74-1.46)

### 3.2 Regional and national levels of CKD-T2D

In 2019, the regions with the highest ASIR of CKD-T2D were North Africa and the Middle East, reporting a rate of 61.33 per 100,000 people (95% UI: 55.98 to 67.44). This was followed by Central Latin America with a rate of 53.46 per 100,000 (95% UI: 49.17 to 58.15), and Southern Latin America with a rate of 38.54 per 100,000 (95% UI: 34.99 to 42.37). Notably, Andean Latin America experienced a substantial increase in ASIR of CKD-T2D, with an estimated annual percentage change (EAPC) of 2.23 (95% CI: 2.11 to 2.34). Similar increases were observed in Central Asia (EAPC = 1.89, 95% CI: 1.72 to 2.06) and North Africa and the Middle East (EAPC = 2.03, 95% CI: 1.92 to 2.14). Conversely, no region showed a noteworthy reduction in ASIR of CKD-T2D, as **Table 1**, **Figure 1**, **Supplementary Figures 1, 2** demonstrate.

The age-standardized DALY rate of CKD-T2D in 2019 varied across different regions. Central Latin America had the highest rate at 320.2 per 100,000 (95% UI 255.6 to 388.56). Southeast Asia followed at 235.7 per 100,000 (95% UI: 194.17 to 281.24), and Oceania at 206.91 per 100,000 (95% UI: 164.32 to 256.32). The North America region showed a noticeable upward trend in rates (EAPC = 2.73, 95% CI: 2.39 to 3.07), while the High-income Asia Pacific region exhibited a significant decrease (EAPC = -0.87, 95% CI: -1.05 to -0.68); these trends can be found in **Table 2**, **Figure 3**, and **Supplementary Figure 1**.

In 2019, the ASIR of CKD-T2D ranged from 13.91 to 78.37 per 100,000, with the highest rates observed in Saudi Arabia and the lowest in the Republic of Moldova (**Supplementary Figure 1**, **Supplementary Table 1**). The DALYs rates for CKD-T2D varied from 18.13 to 733.03 per 100,000. Mauritius had the highest DALY rate, while Iceland had the lowest (**Supplementary Tables 1, 2**, **Supplementary Figure 1**).

From 1990 to 2019, Morocco experienced the largest increase in ASIR for CKD-T2D, with an EAPC of 2.72 (95% CI 2.65 to 2.8). Conversely, Greece showed the largest decrease in ASIR for CKD-T2D, with an EAPC of -0.14 (95% CI -0.27 to -0.01). These findings are depicted in **Supplementary Figure 1** and further supported by the data presented in **Supplementary Table 1, 2**. Furthermore, Armenia exhibited the greatest increase in DALYs rates, with an EAPC of 4.26 (95% CI 3.95 to 4.58). In contrast, Mongolia demonstrated the largest decrease in DALY rates, with an EAPC of -3.18 (95% CI -3.62 to -2.74). These results are illustrated in **Supplementary Figure 1** and corroborated by the information provided in **Supplementary Table 2** and **Table 2**.

### 3.3 Age and sex specific CKD-T2D

The burden of CKD-T2D is particularly prominent among the elderly, especially in the age group of 60-79 years and older. This trend is more noticeable in males, indicating that the health risks associated with CKD-T2D may escalate with increasing age, particularly for older men. These findings suggest that older populations are affected more severely by CKD-T2D, and this impact tends to accelerate after the age of 60-79. The rate of DALYs for CKD-T2D exhibited a significant increase starting at the age of 55 in 2019, accompanied by a noteworthy rise in ASIR for those aged 60-79. It is undeniable that the global burden of CKD-T2D is at its lowest among individuals under 20 years old and reaches its highest level in individuals over 80 years old (**Supplementary Table 3, 4**, **Figure 2**, **Supplementary Figure 3**).

### 3.4 Correlation between socio-demographic index and CKD-T2D burden

**Supplementary Figure 4** demonstrates a significant negative correlation between the SDI and both the ASIR and the age-standardized DALYs rate for CKD-T2D in 2019 at the national level. The correlation coefficients (ρ) are 0.38 (P< 0.001) for the SDI and ASIR, and -0.264 (P< 0.001) for the SDI and DALY rate. This suggests that as socioeconomic status improves, the disease burden is expected to decrease over time, while the ASIR may increase. Generally, countries with higher SDI levels have a lower burden of CKD-T2D measured by the age-standardized DALY rate, with the highest ASIR of 37.74 observed among the five SDI regions (refer to **Supplementary Figure 4**, **Tables 1**, **2**).

The association between SDI and CKD-T2D is intricate and displays notable regional variations. As depicted in **Supplementary Figure 4**, the ASIR and age-standardized DALY rate for CKD-T2D demonstrate an initial increase in countries with an SDI below 0.7. However, as the SDI increases, these rates begin to decline, with the largest burden observed in countries with a moderate SDI range. Notably, over the span of the past three decades, only countries with a low SDI have witnessed a decrease in age-standardized DALY rate for CKD-T2D, whereas countries with a high SDI have experienced the most substantial increases, as evidenced in **Table 2** and **Figure 3**.

Regions with a high SDI demonstrated the highest ASIR for CKD-T2D at 37.74 per 100,000 (95% UI: 34.43 to 41.16), while the lowest ASIR in 2019 was observed in regions with a low SDI, at 20.48 per 100,000 (95% UI: 18.48 to 22.69). Regarding the Age-Standardized DALYs rate for CKD-T2D, there were contrasting patterns compared to ASIR. The middle SDI regions exhibited the highest rate at 159.63 per 100,000 (95% UI: 132.14 to 188.19). Conversely, regions with a high SDI had the lowest age-standardized DALY rate at 80.85 per 100,000 (95% UI: 66.99 to 95.28).

## 4 Discussion

Between 1990 and 2019, there was a clear global increase in the burden of CKD-T2D, as shown by a notable rise in both incidence and DALYs. Our findings demonstrate that CKD is a common complication of T2DM, with the risk being particularly significant among older individuals who are more susceptible to comorbidities. As a result, there is a higher DALY rate among individuals aged 65 and above. We also observed a rising trend in ASRs of CKD-T2D, with a marked increase in incidence among males over 50 years old. This indicates the compounded risks associated with age and gender-specific factors. Previous studies have shown notable racial disparities in diabetes between women and men, with a distinct life course relationship observed in women. Although men had a greater disease incidence in 2019, the increasing rates in women suggest an imminent rise in CKD-T2D incidence among female populations. Contributing factors to this trend include the potential overestimation of disease in women through glomerular filtration rate-estimating equations, age-related decline in protective hormones, and societal factors such as a higher likelihood of women seeking screening or diagnosis (16, 17).

The burden of CKD-T2D varies significantly among countries and regions, and is greatly influenced by levels of social development. Regions with high SDI are particularly affected, showing the highest ASIR. In contrast, countries with lower levels of SDI often face challenges in social progress and healthcare effectiveness, resulting in limited burden of CKD-T2D. These disparities have likely contributed to the increased ASIR of CKD-T2D in high-middle SDI regions (3). The screening for CKD, through regular measurement of the albumin-to-creatinine ratio and glomerular filtration rate, is widely recommended for individuals with type 2 diabetes as part of the annual cycle of care starting from the time of diagnosis (4). Therefore, implementing screening and intervention measures for CKD-T2D in high SDI regions may lead to the highest ASIR and lowest age-standardized DALYs rate. In nations with higher SDI, aging plays a more noteworthy role, whereas in countries with lower SDI, population growth characterized by high fertility and lower life expectancy is a major contributing factor (18).

The main focus of this study is to evaluate the spatial and temporal changes in the burden of CKD-T2D. In previous studies, it was found that the incidence rate, and mortality of CKD-T2D were different in age group and gender. Therefore, in order to control the impact of confounding factors, age and gender specific CKD-T2D burdens were calculated to understand their impact. In addition, age standardization was carried out to reduce the impact of age on the results.

Despite the overall increase in the burden of CKD-T2D, certain regions have made notable progress in preventing and managing this condition, particularly in low- and high-middle SDI regions, where a decrease in age-standardized DALYs rates has been observed. However, the effectiveness of interventions varies significantly between countries. For example, Mauritius and Nauru have demonstrated less favorable outcomes, revealing missed opportunities and emphasizing the need for targeted healthcare strategies. From 1990 to 2019, some countries have made significant advancements that align with rapid socioeconomic development, while others, particularly Mauritius and Nauru, have fallen behind. In 2019, there were evident disparities in DALY rates among countries, highlighting the potential for narrowing these gaps. The impressive performances in some countries across the development spectrum should inspire others with similar SDI to optimize their resources for improved health outcomes. The challenges posed by low socioeconomic development, though substantial, are not insurmountable (19). Health progress, driven by sociodemographic advancement, can be influenced by additional factors. This variability is partially explained by shifts in patterns of risk factors such as hyperglycemia, hypertension, and obesity (20), providing an opportunity to move beyond the notion that managing the burden of kidney disease is solely about addressing diabetes and hypertension. Emerging evidence on the interplay of smoking and ambient pollution with diabetic nephropathy in different regions of the world further underscores this point (21, 22).

Interpretation of these findings requires consideration of certain limitations inherent in the GBD 2019 study. The estimation of CKD burden by GBD relies on statistical methods and predicted covariate values from various sources, including census data, disease registration records, household surveys, health service utilization statistics, air pollution monitoring, and vital statistics. While countries like China, USA, India, Australia, UK, and Russia provide high-quality results through established medical registration systems, there is a lack of large, high-quality, population-based CKD studies in certain countries or territories such as the Cook Islands, Niue, Vatican City, Liechtenstein, Order of Malta, and Palestine (23). This introduces bias to the primary data from these areas. Therefore, caution is needed when extrapolating specific data to WHO non-member countries and regions with underdeveloped medical systems. The limited data also hinders further investigation into the burden of CKD-T2D at different stages, underscoring the need for increased investment in improving vital registration and data collection efforts in developing countries. Despite these limitations, this analysis offers valuable new insights into the global burden of CKD-T2D.

The study period spanning from 1990 to 2019 witnessed a significant rise in the global burden of CKD-T2D. This increase was accurately measured through ASIR and age-standardized DALYs rates. Additionally, we found substantial variations in the demographic and epidemiological trends across different levels of SDI regions. Although we observed a negative correlation between socioeconomic status and disease burden, countries demonstrated a wide range of performance. While some countries surpassed expectations in managing the disease burden, others experienced a rise in DALY rates despite sociodemographic advancements. With limited resources available, it is crucial to prioritize early-stage interventions and focus on establishing causal pathways to alleviate the death burden in low-to-middle SDI countries. Furthermore, the increasing public health concerns related to overweight and obesity, coupled with CKD resulting from type 2 diabetes mellitus, call for immediate implementation of tailored prevention programs. Such programs may include interventions like diet modification or physical activity initiatives, particularly in high-income countries.

## 5 Conclusion

From 1990 to 2019, there were notable regional and national variations in the increasing burden of CKD-T2D. All age-standarized rates of CKD-T2D showed a consistent upward trend, with higher rates among males compared to females, starting at the age of 60. The majority of the global burden was concentrated in countries with a middle SDI. Central Latin America, followed closely by Southeast Asia and Andean Latin America, experienced the most significant impact of CKD-T2D. Among countries, Mauritius, Palau, and Nicaragua had the highest disease burdens. These findings are crucial for directing epidemiological surveillance efforts and designing targeted health interventions. Therefore, it is important to enhance disease detection strategies and develop tailored early intervention approaches that reflect the varying levels of socioeconomic development. This approach could effectively reduce the burden of CKD-T2D. In conclusion, these findings highlight the need for strategic planning in healthcare policies to address the changing landscape of CKD-T2D.

## Data availability statement

The original contributions presented in the study are included in the article/**Supplementary Material**. Further inquiries can be directed to the corresponding author.

## Ethics statement

The studies involving humans were approved by Beilun District People’s Hospital. The studies were conducted in accordance with the local legislation and institutional requirements. The participants provided their written informed consent to participate in this study.

## Author contributions

XD: Writing – original draft, Writing – review & editing. XL: Writing – original draft, Writing – review & editing. YY: Writing – original draft, Writing – review & editing. JJ: Writing – original draft, Writing – review & editing. ML: Writing – original draft, Writing – review & editing. LS: Writing – original draft, Writing – review & editing.
