# Supplementary figures and images for: Epidemiological patterns of chronic kidney disease attributed to type 2 diabetes from 1990-2019
Source: Front Endocrinol (Lausanne). 2024 Apr 17;15:1383777. doi: 10.3389/fendo.2024.1383777 (PMC11061475; doi:10.3389/fendo.2024.1383777)

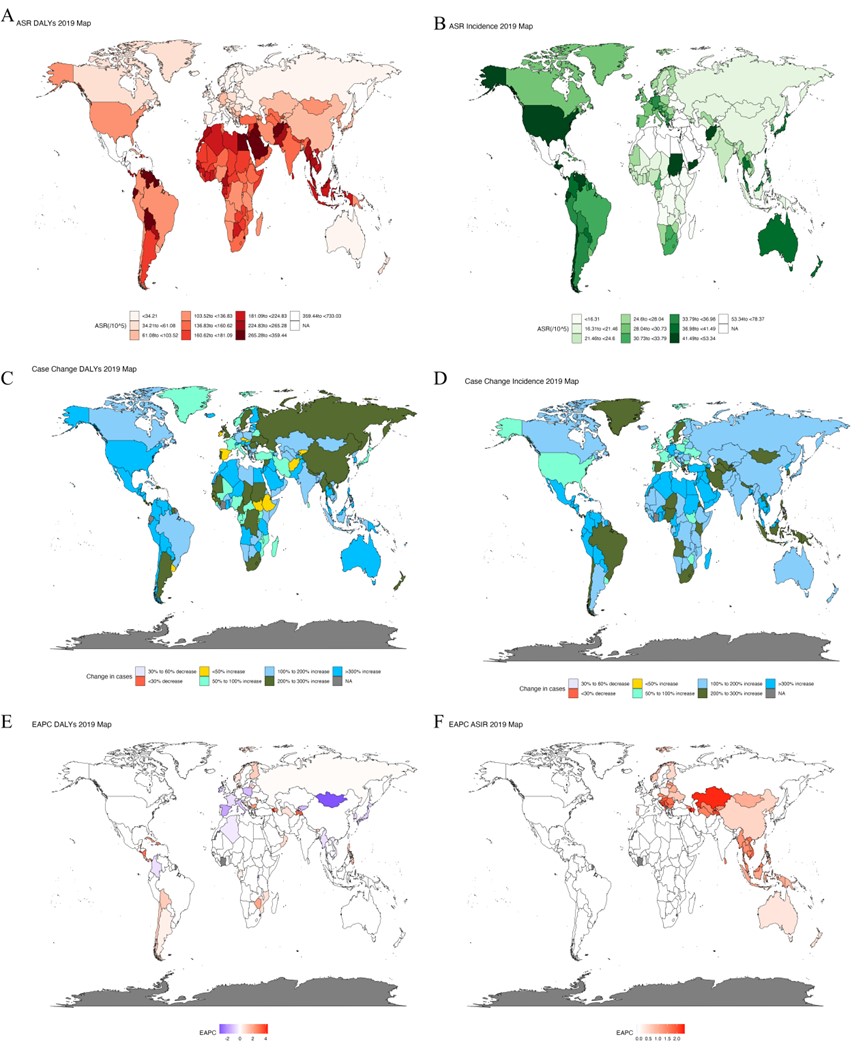

Supplement: Supplementary file 1 [file Image_1.jpg]

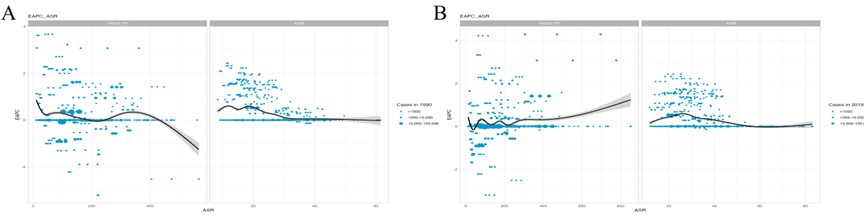

Supplement: Supplementary file 2 [file Image_2.jpg]

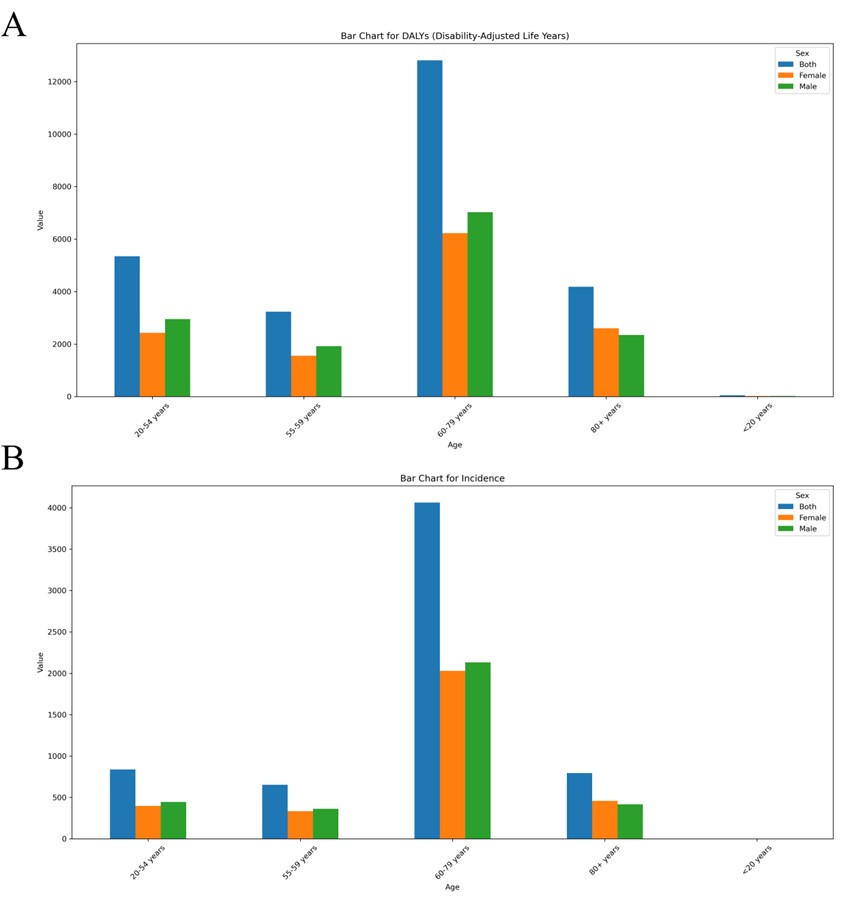

Supplement: Supplementary file 3 [file Image_3.jpg]

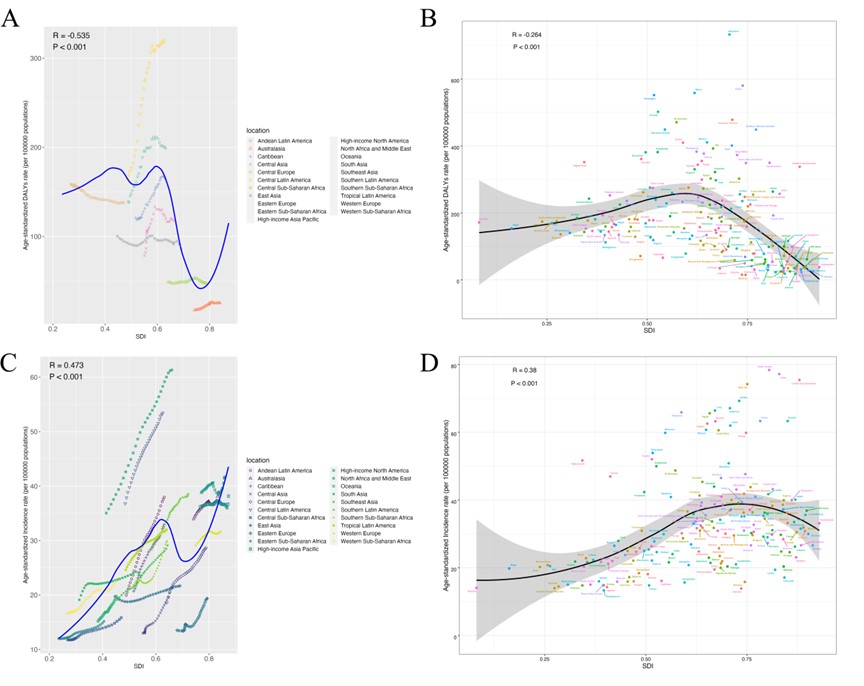

Supplement: Supplementary file 4 [file Image_4.jpg]
