# Supplementary material for: Epidemiological patterns of chronic kidney disease attributed to type 2 diabetes from 1990-2019
Source: Front Endocrinol (Lausanne). 2024 Apr 17;15:1383777. doi: 10.3389/fendo.2024.1383777 (PMC11061475; doi:10.3389/fendo.2024.1383777)
Supplement: Supplementary file 5 [file Table_1.docx]

**Supplementary Table 1.** Incidence of Chronic kidney disease due to type 2 diabetes mellitus in 1990 and 2019 for both sexes and all nations.

| Location | Numbers (95%UI)_1990 | Age-standardized rate (95%UI)_1990 | Numbers (95%UI)_2019 | Age-standardized rate (95%UI)_2019 | Estimated annual percentage changes (95%CI) | Numbers_  change (95%UI) |
| --- | --- | --- | --- | --- | --- | --- |
| Afghanistan | 2331 (2032-2670) | 32.04 (28.13-36.52) | 6084 (5339-6990) | 51.7 (45.52-58.89) | 1.76  (1.67-1.85) | 1.61 (1.42-1.81) |
| Albania | 337 (292-386) | 16.09 (14.08-18.41) | 1133 (987-1281) | 25.77 (22.49-29.09) | 1.63  (1.59-1.68) | 2.36 (2.03-2.71) |
| Algeria | 4625 (4027-5285) | 36.45 (32.3-41.18) | 21234 (18647-24141) | 62.55 (55.17-70.39) | 1.88  (1.76-1.99) | 3.59 (3.34-3.85) |
| American Samoa | 7 (6-8) | 28.3 (24.79-32.2) | 20 (17-22) | 40.02 (35.36-44.87) | 1.11  (0.99-1.22) | 1.92 (1.75-2.13) |
| Andorra | 17 (14-19) | 29.93 (26.45-33.73) | 42 (37-47) | 30.83 (27.05-34.65) | 0.05  (-0.07-0.17) | 1.55 (1.39-1.73) |
| Angola | 419 (371-479) | 11.12 (9.96-12.39) | 1728 (1532-1979) | 15.99 (14.22-18.07) | 1.32  (1.21-1.44) | 3.12 (2.92-3.32) |
| Antigua and Barbuda | 14 (12-16) | 27.45 (24.32-30.84) | 44 (39-50) | 41.84 (36.79-46.66) | 1.43  (1.3-1.56) | 2.17 (1.97-2.39) |
| Argentina | 9383 (8226-10487) | 28.76 (25.43-32.02) | 20262 (18182-22532) | 36.97 (33.23-41.07) | 0.81  (0.71-0.91) | 1.16 (0.99-1.36) |
| Armenia | 320 (270-381) | 11.5 (9.87-13.41) | 927 (806-1069) | 21.95 (19.12-25.09) | 2.42  (2.3-2.53) | 1.89 (1.62-2.21) |
| Australia | 7005 (6492-7576) | 34.2 (31.8-36.89) | 16522 (14834-18270) | 38.62 (34.72-42.61) | 0.33  (0.26-0.39) | 1.36 (1.2-1.54) |
| Austria | 3585 (3103-4091) | 28.82 (24.99-32.72) | 6584 (5812-7450) | 36.27 (32-40.42) | 0.68  (0.6-0.75) | 0.84 (0.7-1) |
| Azerbaijan | 660 (558-782) | 12.69 (10.94-14.85) | 2434 (2078-2821) | 23.48 (20.55-26.73) | 2.2  (1.98-2.43) | 2.69 (2.38-3.07) |
| Bahamas | 39 (34-44) | 24.59 (21.6-27.69) | 143 (127-162) | 35.19 (31.35-39.56) | 1.15  (0.99-1.3) | 2.68 (2.45-2.94) |
| Bahrain | 78 (68-89) | 43.6 (39.02-49.09) | 826 (703-967) | 74.22 (66.4-82.63) | 1.92  (1.76-2.07) | 9.66 (8.9-10.46) |
| Bangladesh | 5608 (4800-6434) | 12.09 (10.5-13.81) | 24345 (20957-27864) | 18.42 (15.92-21.01) | 1.41  (1.24-1.59) | 3.34 (3.04-3.63) |
| Barbados | 66 (58-74) | 24.05 (21.09-27.21) | 178 (159-204) | 36.3 (32.27-41.44) | 1.34  (1.21-1.48) | 1.7 (1.52-1.91) |
| Belarus | 1554 (1350-1808) | 12.04 (10.46-13.9) | 2462 (2157-2813) | 15.82 (13.85-17.97) | 0.99  (0.68-1.29) | 0.58 (0.45-0.76) |
| Belgium | 5038 (4473-5624) | 31.28 (27.91-34.9) | 7642 (6905-8430) | 33.23 (29.97-36.91) | 0.21  (0.15-0.28) | 0.52 (0.36-0.66) |
| Belize | 22 (19-26) | 23.8 (20.58-27.27) | 105 (92-119) | 36.63 (32.37-40.8) | 1.43  (1.36-1.5) | 3.74 (3.33-4.18) |
| Benin | 340 (302-382) | 17.08 (15.27-19.11) | 1094 (958-1243) | 22.83 (20.11-25.78) | 1.01  (0.95-1.08) | 2.21 (2.06-2.38) |
| Bermuda | 15 (13-17) | 23.99 (21.12-27.06) | 48 (43-54) | 38.25 (34.05-42.97) | 1.58  (1.38-1.77) | 2.16 (1.96-2.4) |
| Bhutan | 40 (34-47) | 15.69 (13.63-18.01) | 144 (125-165) | 24.97 (21.9-28.4) | 1.76  (1.7-1.82) | 2.56 (2.35-2.78) |
| Bolivia (Plurinational State of) | 711 (629-801) | 22.16 (19.76-24.73) | 3043 (2696-3390) | 34.27 (30.56-38.13) | 1.49  (1.44-1.53) | 3.28 (3.05-3.53) |
| Bosnia and Herzegovina | 703 (619-801) | 16.65 (14.8-18.86) | 1807 (1590-2043) | 29.55 (26.13-33.13) | 2.23  (2.09-2.36) | 1.57 (1.34-1.83) |
| Botswana | 116 (100-132) | 19.95 (17.52-22.47) | 428 (372-486) | 31.01 (27.31-34.89) | 1.41  (1.24-1.57) | 2.7 (2.52-2.91) |
| Brazil | 22457 (20171-24912) | 24.64 (22.16-27.37) | 76106 (69140-84244) | 31.84 (28.99-35.17) | 0.81  (0.75-0.88) | 2.39 (2.24-2.57) |
| Brunei Darussalam | 39 (34-44) | 43.09 (38.02-48.31) | 132 (113-154) | 45.99 (40.09-52.3) | 0.18  (0.05-0.3) | 2.38 (2.2-2.56) |
| Bulgaria | 2364 (2043-2783) | 18.32 (15.97-21.21) | 4214 (3710-4776) | 29.65 (26.28-33.47) | 1.65  (1.6-1.7) | 0.78 (0.59-1) |
| Burkina Faso | 672 (591-753) | 15.25 (13.56-16.92) | 1866 (1631-2098) | 20.81 (18.29-23.26) | 1.15  (1.06-1.24) | 1.78 (1.64-1.96) |
| Burundi | 273 (243-305) | 11.77 (10.54-13.06) | 631 (555-718) | 14.49 (12.88-16.24) | 0.82  (0.75-0.9) | 1.32 (1.19-1.46) |
| Cabo Verde | 33 (29-37) | 14.12 (12.37-15.82) | 93 (82-106) | 23.07 (20.24-26.02) | 1.91  (1.79-2.02) | 1.86 (1.69-2.06) |
| Cambodia | 647 (558-750) | 14.09 (12.28-16.16) | 2694 (2354-3071) | 22.12 (19.43-24.94) | 1.56  (1.45-1.66) | 3.16 (2.9-3.45) |
| Cameroon | 981 (865-1116) | 22.09 (19.72-24.89) | 3710 (3264-4226) | 30.95 (27.39-35.09) | 1.25  (1.17-1.33) | 2.78 (2.59-2.99) |
| Canada | 9866 (8487-11199) | 29.46 (25.51-33.43) | 21450 (18872-24127) | 30.51 (26.88-34.26) | 0.26  (0.2-0.31) | 1.17 (0.91-1.42) |
| Central African Republic | 129 (111-148) | 11.11 (9.86-12.45) | 294 (255-338) | 13.99 (12.34-15.7) | 0.85  (0.76-0.94) | 1.28 (1.18-1.39) |
| Chad | 454 (402-511) | 15.89 (14.17-17.73) | 1116 (985-1275) | 20.24 (17.99-22.93) | 0.86  (0.74-0.97) | 1.46 (1.33-1.58) |
| Chile | 2859 (2533-3239) | 29.14 (25.9-32.94) | 10633 (9524-11898) | 43.81 (39.27-49.05) | 1.44  (1.33-1.56) | 2.72 (2.43-3.07) |
| China | 163054 (144123-183482) | 19.44 (17.31-21.78) | 433952 (390222-481041) | 21.29 (19.23-23.64) | 0.53  (0.45-0.62) | 1.66 (1.56-1.76) |
| Colombia | 5340 (4673-6129) | 29.78 (26.16-33.94) | 21753 (19386-24599) | 41.32 (36.78-46.76) | 1.01  (0.94-1.09) | 3.07 (2.71-3.47) |
| Comoros | 27 (24-31) | 12.39 (11.03-13.85) | 76 (67-86) | 15.93 (14.14-17.86) | 0.94  (0.88-1.01) | 1.78 (1.63-1.95) |
| Congo | 136 (118-157) | 12.67 (11.17-14.23) | 457 (397-520) | 17.84 (15.68-20.1) | 1.34  (1.23-1.46) | 2.37 (2.2-2.53) |
| Cook Islands | 3 (3-4) | 24.17 (21.17-27.25) | 9 (8-10) | 36.4 (31.84-41.03) | 1.26  (1.17-1.35) | 1.91 (1.74-2.1) |
| Costa Rica | 1066 (986-1156) | 60.77 (56.43-65.37) | 3201 (2976-3459) | 60.6 (56.57-65.33) | -0.05  (-0.06--0.03) | 2 (1.94-2.08) |
| Côte d'Ivoire | 768 (680-870) | 19.47 (17.4-21.64) | 2558 (2260-2905) | 24.58 (21.94-27.49) | 0.86  (0.78-0.94) | 2.33 (2.14-2.49) |
| Croatia | 1451 (1262-1675) | 22.13 (19.28-25.32) | 2981 (2649-3354) | 34.52 (30.72-38.64) | 1.64  (1.56-1.72) | 1.05 (0.84-1.33) |
| Cuba | 1859 (1636-2115) | 18.12 (15.98-20.56) | 5914 (5206-6694) | 31.78 (27.96-36) | 1.9  (1.82-1.99) | 2.18 (1.92-2.45) |
| Cyprus | 340 (291-388) | 38.36 (33.63-42.93) | 824 (718-937) | 39.96 (35.2-45.19) | 0.14  (0.01-0.26) | 1.42 (1.28-1.57) |
| Czechia | 2617 (2275-2967) | 18.91 (16.52-21.37) | 6072 (5279-6875) | 28.79 (25.15-32.39) | 1.36  (1.25-1.47) | 1.32 (1.14-1.51) |
| Democratic People's Republic of Korea | 3027 (2643-3458) | 19.11 (16.89-21.61) | 7543 (6634-8521) | 23.41 (20.65-26.51) | 0.72  (0.54-0.9) | 1.49 (1.34-1.67) |
| Democratic Republic of the Congo | 1853 (1618-2112) | 11.8 (10.55-13.15) | 5322 (4684-6010) | 15.49 (13.74-17.25) | 0.93  (0.84-1.02) | 1.87 (1.71-2.04) |
| Denmark | 2167 (1872-2466) | 25.74 (22.35-29.18) | 3824 (3361-4296) | 31.86 (28.18-35.55) | 0.8  (0.76-0.83) | 0.76 (0.6-0.93) |
| Djibouti | 17 (15-19) | 12.59 (11.22-14.09) | 100 (87-113) | 17.25 (15.33-19.34) | 1.21  (1.09-1.33) | 4.92 (4.62-5.25) |
| Dominica | 20 (18-23) | 29.21 (25.68-32.94) | 35 (31-39) | 39.2 (34.65-43.99) | 0.95  (0.79-1.12) | 0.75 (0.63-0.85) |
| Dominican Republic | 614 (535-709) | 16.09 (14.14-18.42) | 2701 (2386-3063) | 28.72 (25.47-32.37) | 1.74  (1.61-1.88) | 3.4 (3.11-3.72) |
| Ecuador | 1181 (1027-1346) | 22.3 (19.46-25.42) | 6925 (6169-7739) | 45.36 (40.59-50.56) | 2.4  (2.25-2.54) | 4.86 (4.39-5.36) |
| Egypt | 10648 (9371-12229) | 36.47 (32.24-41.62) | 43073 (37762-49122) | 65.61 (58.36-73.72) | 2  (1.87-2.13) | 3.05 (2.87-3.22) |
| El Salvador | 895 (771-1045) | 29.79 (25.76-34.73) | 2823 (2510-3179) | 48.71 (43.18-54.9) | 1.71  (1.63-1.79) | 2.16 (1.93-2.43) |
| Equatorial Guinea | 21 (19-24) | 11.03 (9.85-12.29) | 93 (82-106) | 20 (17.75-22.47) | 2.36  (2.22-2.5) | 3.39 (3.14-3.66) |
| Eritrea | 101 (88-118) | 10.69 (9.42-12.07) | 384 (335-441) | 14.87 (13.06-16.8) | 1.08  (0.99-1.16) | 2.78 (2.58-3) |
| Estonia | 302 (261-355) | 14.78 (12.72-17.27) | 593 (510-687) | 24.8 (21.3-28.6) | 1.97  (1.81-2.13) | 0.96 (0.81-1.12) |
| Eswatini | 69 (60-79) | 23.36 (20.58-26.57) | 180 (157-207) | 30.28 (26.73-34.1) | 0.76  (0.55-0.96) | 1.61 (1.49-1.75) |
| Ethiopia | 2313 (2042-2592) | 11.89 (10.65-13.19) | 5878 (5211-6569) | 14.79 (13.16-16.53) | 0.7  (0.61-0.79) | 1.54 (1.44-1.65) |
| Fiji | 98 (84-112) | 26 (22.77-29.29) | 263 (227-298) | 33.66 (29.57-37.42) | 0.75  (0.7-0.79) | 1.69 (1.46-1.9) |
| Finland | 1504 (1299-1737) | 20.21 (17.59-23.02) | 3160 (2777-3570) | 24.6 (21.79-27.66) | 0.5  (0.41-0.58) | 1.1 (0.92-1.3) |
| France | 22876 (20396-25855) | 26.27 (23.36-29.6) | 40875 (36345-45861) | 29.73 (26.3-33.47) | 0.3  (0.23-0.37) | 0.79 (0.64-0.93) |
| Gabon | 85 (74-96) | 15.07 (13.33-16.76) | 237 (206-268) | 22.98 (20.22-25.7) | 1.51  (1.45-1.58) | 1.78 (1.62-1.93) |
| Gambia | 59 (52-66) | 16.7 (14.9-18.63) | 210 (187-235) | 22.34 (19.92-24.91) | 0.99  (0.94-1.05) | 2.59 (2.41-2.77) |
| Georgia | 792 (674-933) | 12.56 (10.84-14.63) | 1168 (1029-1338) | 20.38 (18-23.48) | 1.53  (1.41-1.64) | 0.47 (0.33-0.64) |
| Germany | 42200 (37449-47299) | 32.39 (28.7-36.23) | 70550 (64278-77317) | 36.87 (33.76-40.04) | 0.3  (0.24-0.37) | 0.67 (0.55-0.82) |
| Ghana | 915 (800-1042) | 14.74 (13.03-16.6) | 3440 (3006-3885) | 21.62 (19.04-24.17) | 1.31  (1.24-1.37) | 2.76 (2.56-2.96) |
| Greece | 6030 (5275-6789) | 37.96 (33.29-42.65) | 8538 (7595-9489) | 36.26 (31.98-40.37) | -0.14  (-0.27--0.01) | 0.42 (0.33-0.51) |
| Greenland | 7 (6-8) | 23.95 (21.08-26.97) | 20 (18-23) | 30.26 (26.81-33.94) | 0.76  (0.72-0.79) | 1.9 (1.71-2.14) |
| Grenada | 18 (16-20) | 26.8 (23.64-30.09) | 51 (45-58) | 43.96 (38.72-49.18) | 1.55  (1.41-1.7) | 1.82 (1.64-2.04) |
| Guam | 19 (16-22) | 23.27 (20.25-26.48) | 60 (52-68) | 31.1 (27.23-35.45) | 0.77  (0.6-0.93) | 2.16 (1.95-2.38) |
| Guatemala | 1212 (1060-1397) | 31.71 (27.91-36.24) | 5601 (4973-6378) | 49.98 (44.31-56.69) | 1.5  (1.39-1.61) | 3.62 (3.35-3.92) |
| Guinea | 554 (488-625) | 16.51 (14.73-18.56) | 1186 (1046-1351) | 21.85 (19.38-24.8) | 0.98  (0.91-1.05) | 1.14 (1.03-1.27) |
| Guinea-Bissau | 73 (64-82) | 17.78 (16-19.78) | 155 (136-179) | 21.63 (19.13-24.4) | 0.72  (0.63-0.81) | 1.13 (1.02-1.25) |
| Guyana | 89 (78-102) | 22.43 (19.9-25.27) | 239 (209-272) | 35.86 (31.67-40.41) | 1.37  (1.27-1.47) | 1.67 (1.5-1.85) |
| Haiti | 664 (575-767) | 19.53 (17.2-22.18) | 2027 (1765-2313) | 27.94 (24.53-31.81) | 1.18  (1.15-1.22) | 2.06 (1.92-2.2) |
| Honduras | 635 (548-730) | 29.81 (25.89-34.11) | 2664 (2340-3028) | 42.34 (37.24-48.05) | 1.14  (1.1-1.17) | 3.2 (2.96-3.45) |
| Hungary | 2376 (2075-2709) | 15.92 (13.95-18.12) | 5620 (4924-6395) | 29.2 (25.77-33.09) | 1.99  (1.87-2.11) | 1.37 (1.2-1.55) |
| Iceland | 72 (64-81) | 24.57 (21.97-27.64) | 146 (129-164) | 26.24 (23.18-29.35) | -0.02  (-0.16-0.13) | 1.04 (0.89-1.22) |
| India | 95773 (84971-107758) | 20 (17.92-22.37) | 278934 (248867-310804) | 24.06 (21.54-26.69) | 0.43  (0.32-0.54) | 1.91 (1.79-2.04) |
| Indonesia | 15208 (13505-17146) | 15.07 (13.47-16.87) | 48065 (42760-54217) | 21.46 (19.33-24) | 1.08  (0.99-1.17) | 2.16 (2.07-2.26) |
| Iran (Islamic Republic of) | 11475 (9992-13063) | 41.75 (37.27-46.62) | 40490 (36550-44831) | 55.56 (50.11-61.29) | 1.01  (0.91-1.11) | 2.53 (2.34-2.72) |
| Iraq | 3153 (2769-3563) | 40.64 (35.78-45.92) | 15499 (13642-17454) | 66.41 (59.41-74.19) | 1.86  (1.8-1.92) | 3.92 (3.7-4.14) |
| Ireland | 1489 (1328-1629) | 34.27 (30.64-37.41) | 2543 (2425-2662) | 33.49 (31.95-35.1) | -0.06  (-0.09--0.03) | 0.71 (0.57-0.9) |
| Israel | 1918 (1667-2138) | 37.77 (33.08-41.99) | 4861 (4274-5429) | 41.74 (36.94-46.45) | 0.32  (0.19-0.45) | 1.53 (1.34-1.73) |
| Italy | 26277 (23265-29622) | 28.22 (24.98-31.73) | 42915 (38488-47598) | 29 (26.08-32.2) | 0.03  (-0.02-0.08) | 0.63 (0.54-0.72) |
| Jamaica | 445 (391-508) | 25.6 (22.41-29.12) | 1009 (883-1144) | 34.2 (29.89-38.84) | 1.02  (0.83-1.21) | 1.27 (1.12-1.41) |
| Japan | 64964 (59003-71970) | 37.53 (34.2-41.45) | 136480 (122938-150493) | 40.12 (36.2-44.31) | 0.04  (-0.01-0.1) | 1.1 (1-1.19) |
| Jordan | 544 (477-613) | 40.46 (35.83-45.29) | 4693 (4172-5242) | 69.27 (62.34-76.89) | 2  (1.91-2.1) | 7.63 (7.06-8.18) |
| Kazakhstan | 1553 (1312-1820) | 11.82 (10.09-13.68) | 3868 (3296-4547) | 21.04 (18.2-24.28) | 2.3  (2.04-2.57) | 1.49 (1.34-1.65) |
| Kenya | 941 (841-1053) | 11.77 (10.53-13.09) | 3151 (2786-3547) | 14.8 (13.2-16.6) | 0.69  (0.6-0.78) | 2.35 (2.28-2.42) |
| Kiribati | 7 (6-8) | 17.96 (15.65-20.65) | 18 (15-21) | 24.63 (21.53-28.01) | 0.98  (0.91-1.05) | 1.5 (1.35-1.66) |
| Kuwait | 259 (223-294) | 41.56 (36.93-46.87) | 1628 (1412-1868) | 63.32 (56.02-71.32) | 1.54  (1.48-1.6) | 5.29 (4.64-5.98) |
| Kyrgyzstan | 336 (282-394) | 10.67 (9.07-12.37) | 784 (660-925) | 15.67 (13.46-18.22) | 1.48  (1.25-1.71) | 1.34 (1.15-1.54) |
| Lao People's Democratic Republic | 448 (389-511) | 20.71 (18.26-23.34) | 1332 (1165-1515) | 29.37 (25.87-33.1) | 1.19  (1.09-1.28) | 1.97 (1.84-2.14) |
| Latvia | 446 (386-515) | 12.54 (10.84-14.45) | 703 (615-804) | 19.41 (16.87-22.26) | 1.46  (1.26-1.66) | 0.58 (0.46-0.71) |
| Lebanon | 847 (742-961) | 36.47 (32.16-41.08) | 3478 (3130-3873) | 67.11 (60.1-75.06) | 2.24  (2.06-2.42) | 3.1 (2.82-3.41) |
| Lesotho | 191 (168-216) | 19.2 (17.06-21.52) | 328 (287-374) | 25.42 (22.62-28.7) | 0.8  (0.57-1.03) | 0.72 (0.63-0.82) |
| Liberia | 188 (164-213) | 16.43 (14.52-18.36) | 442 (387-506) | 22.31 (19.66-25.25) | 1.14  (1.02-1.26) | 1.35 (1.2-1.51) |
| Libya | 687 (607-781) | 37.16 (33.05-42.06) | 3065 (2716-3428) | 61.98 (55.25-69.59) | 1.9  (1.74-2.06) | 3.46 (3.24-3.68) |
| Lithuania | 615 (531-712) | 13.68 (11.77-15.82) | 899 (789-1021) | 17.14 (15.06-19.62) | 0.81  (0.64-0.98) | 0.46 (0.32-0.63) |
| Luxembourg | 176 (156-197) | 30.77 (27.27-34.43) | 352 (316-389) | 35.62 (31.78-39.68) | 0.51  (0.39-0.62) | 1.01 (0.87-1.17) |
| Madagascar | 534 (472-610) | 10.63 (9.44-11.95) | 1397 (1226-1580) | 13.34 (11.79-15.02) | 0.72  (0.65-0.8) | 1.61 (1.47-1.76) |
| Malawi | 479 (420-543) | 12.66 (11.26-14.17) | 1125 (994-1267) | 16 (14.2-17.96) | 0.89  (0.83-0.95) | 1.35 (1.2-1.5) |
| Malaysia | 2153 (1896-2433) | 23.04 (20.47-25.94) | 9844 (8815-11076) | 35.16 (31.67-39.36) | 1.51  (1.43-1.59) | 3.57 (3.27-3.91) |
| Maldives | 24 (21-28) | 26.11 (22.58-29.71) | 124 (108-140) | 40.9 (36.04-45.89) | 1.7  (1.59-1.81) | 4.1 (3.65-4.66) |
| Mali | 648 (529-810) | 15.3 (12.73-18.85) | 1785 (1459-2247) | 20.52 (17.05-25.82) | 1.05  (0.95-1.15) | 1.75 (1.56-1.94) |
| Malta | 147 (130-166) | 33.73 (29.85-37.82) | 350 (306-391) | 35.89 (31.71-39.91) | 0.18  (0.09-0.27) | 1.37 (1.21-1.57) |
| Marshall Islands | 4 (3-4) | 21.07 (18.56-23.97) | 11 (9-13) | 29.94 (26.64-34.19) | 1.14  (1.11-1.18) | 1.97 (1.8-2.15) |
| Mauritania | 185 (163-211) | 18.17 (16.18-20.47) | 520 (453-588) | 25 (21.84-28.25) | 1.08  (1.04-1.12) | 1.8 (1.66-1.96) |
| Mauritius | 259 (223-299) | 33.49 (28.9-38.01) | 948 (817-1073) | 50.84 (44.04-57.09) | 1.33  (1.22-1.43) | 2.65 (2.21-3.12) |
| Mexico | 18224 (16229-20697) | 41.25 (36.78-46.69) | 73563 (67531-79960) | 60.72 (55.88-65.94) | 1.21  (1.12-1.3) | 3.04 (2.76-3.34) |
| Micronesia (Federated States of) | 12 (10-14) | 23.92 (20.83-27.11) | 27 (23-32) | 36.36 (32.15-41.12) | 1.35  (1.3-1.4) | 1.33 (1.18-1.5) |
| Monaco | 20 (17-22) | 26.3 (23.2-29.67) | 29 (26-33) | 30 (26.56-33.76) | 0.45  (0.42-0.48) | 0.49 (0.39-0.61) |
| Mongolia | 181 (154-213) | 16.42 (14.18-19.12) | 534 (461-627) | 20.8 (18.19-24.15) | 1.02  (0.9-1.14) | 1.96 (1.71-2.23) |
| Montenegro | 157 (136-179) | 25.03 (21.79-28.34) | 357 (314-406) | 35.37 (31.12-40.16) | 1.31  (1.27-1.35) | 1.28 (1.1-1.46) |
| Morocco | 3862 (3366-4459) | 28.19 (24.79-32.25) | 19008 (16750-21608) | 59.83 (53.32-67.35) | 2.72  (2.65-2.8) | 3.92 (3.68-4.2) |
| Mozambique | 642 (570-719) | 11.07 (9.96-12.25) | 1585 (1391-1793) | 15.03 (13.3-16.94) | 1.01  (0.97-1.05) | 1.47 (1.35-1.6) |
| Myanmar | 4481 (3945-5086) | 18.66 (16.58-20.92) | 13212 (11584-14973) | 27.78 (24.53-31.19) | 1.35  (1.24-1.46) | 1.95 (1.79-2.11) |
| Namibia | 144 (126-164) | 19.47 (17.32-21.79) | 376 (330-429) | 26.92 (23.81-30.4) | 1.14  (0.93-1.35) | 1.61 (1.47-1.73) |
| Nauru | 1 (1-1) | 24.18 (21.17-27.79) | 1 (1-2) | 33.05 (28.94-37.69) | 0.93  (0.85-1.01) | 0.54 (0.46-0.62) |
| Nepal | 1557 (1348-1835) | 15.96 (13.97-18.28) | 6168 (5411-7193) | 26.5 (23.31-30.57) | 1.48  (1.4-1.57) | 2.96 (2.76-3.19) |
| Netherlands | 5944 (5416-6551) | 28.39 (25.97-31.19) | 11258 (9990-12535) | 31.56 (28-34.97) | 0.41  (0.36-0.45) | 0.89 (0.73-1.06) |
| New Zealand | 1340 (1188-1505) | 32.77 (29.25-36.55) | 2969 (2665-3344) | 37.23 (33.58-41.91) | 0.38  (0.33-0.43) | 1.22 (1.05-1.43) |
| Nicaragua | 573 (494-663) | 35.05 (30.43-40.21) | 2598 (2257-2941) | 53.9 (47.49-60.56) | 1.45  (1.41-1.49) | 3.53 (3.25-3.82) |
| Niger | 403 (355-457) | 14.88 (13.16-16.58) | 1528 (1339-1757) | 19.82 (17.48-22.45) | 1.08  (1-1.15) | 2.79 (2.6-3.01) |
| Nigeria | 7225 (6472-8016) | 16.61 (14.89-18.37) | 18458 (16515-20531) | 22.14 (19.77-24.65) | 1.16  (1.03-1.29) | 1.55 (1.49-1.62) |
| Niue | 1 (0-1) | 24.7 (21.34-28.2) | 1 (1-1) | 37.2 (32.54-42.35) | 1.33  (1.2-1.46) | 0.55 (0.48-0.64) |
| North Macedonia | 376 (330-424) | 19.6 (17.22-21.95) | 1195 (1063-1348) | 35.21 (31.5-39.58) | 1.96  (1.85-2.08) | 2.18 (1.89-2.47) |
| Northern Mariana Islands | 6 (5-8) | 32.73 (28.21-37.76) | 24 (20-28) | 40.99 (35.94-46.73) | 0.6  (0.37-0.84) | 2.71 (2.25-3.28) |
| Norway | 1551 (1342-1757) | 21.11 (18.62-23.68) | 2620 (2332-2927) | 26.62 (23.81-29.77) | 0.72  (0.64-0.79) | 0.69 (0.58-0.81) |
| Oman | 204 (178-233) | 31.33 (27.56-35.32) | 1113 (974-1270) | 63.31 (56.62-70.92) | 2.35  (2.15-2.56) | 4.46 (4.1-4.82) |
| Pakistan | 10527 (9332-11940) | 18.11 (16.16-20.63) | 26899 (23796-30525) | 23.78 (21.23-26.77) | 1.02  (0.98-1.07) | 1.56 (1.46-1.65) |
| Palau | 3 (2-3) | 28.08 (23.74-32.81) | 9 (8-11) | 39.39 (34.49-45.01) | 0.98  (0.59-1.38) | 2.15 (1.92-2.43) |
| Palestine | 360 (314-407) | 41.06 (36.04-45.91) | 1578 (1384-1788) | 65.89 (58.62-73.69) | 1.56  (1.48-1.64) | 3.38 (3.16-3.63) |
| Panama | 490 (424-564) | 32.49 (28.31-37.42) | 1921 (1722-2154) | 46.5 (41.64-51.99) | 1.07  (0.99-1.16) | 2.92 (2.51-3.4) |
| Papua New Guinea | 212 (179-248) | 10.85 (9.42-12.49) | 730 (621-847) | 14.81 (12.73-17) | 0.89  (0.73-1.05) | 2.44 (2.24-2.67) |
| Paraguay | 551 (484-634) | 24.6 (21.63-28.14) | 2208 (1942-2470) | 39.26 (34.57-43.83) | 1.47  (1.41-1.53) | 3.01 (2.73-3.34) |
| Peru | 2145 (1899-2410) | 18.3 (16.2-20.54) | 11169 (9932-12566) | 35.47 (31.43-39.8) | 2.33  (2.21-2.44) | 4.21 (3.83-4.62) |
| Philippines | 7222 (6514-7960) | 23.81 (21.55-26.12) | 27385 (24698-30237) | 33.68 (30.46-37.06) | 1.07  (1.04-1.11) | 2.79 (2.72-2.87) |
| Poland | 9140 (7895-10618) | 20.63 (17.93-23.89) | 19118 (16668-21953) | 27.29 (23.93-31.3) | 0.28  (0.05-0.51) | 1.09 (0.98-1.21) |
| Portugal | 4018 (3469-4603) | 27.45 (24.03-31.26) | 7622 (6678-8588) | 30.97 (26.85-35.07) | 0.35  (0.27-0.43) | 0.9 (0.78-1.04) |
| Puerto Rico | 1075 (948-1215) | 29.49 (26.13-33.29) | 2831 (2487-3198) | 43.23 (37.78-48.6) | 1.35  (1.14-1.56) | 1.63 (1.41-1.9) |
| Qatar | 52 (45-60) | 46.62 (41.4-52.74) | 868 (728-1027) | 77.18 (68.98-86.16) | 1.79  (1.61-1.98) | 15.66 (14.44-16.86) |
| Republic of Korea | 8014 (6977-9231) | 28.61 (25.21-32.59) | 29455 (26594-32190) | 32.36 (29.26-35.37) | 0.39  (0.31-0.47) | 2.68 (2.3-3.06) |
| Republic of Moldova | 419 (360-486) | 9.54 (8.28-11.05) | 797 (689-929) | 13.91 (12.04-16.16) | 1.04  (0.82-1.25) | 0.9 (0.76-1.07) |
| Romania | 4489 (3938-5103) | 15.71 (13.94-17.88) | 9736 (8690-10883) | 26.63 (23.86-29.58) | 1.73  (1.59-1.87) | 1.17 (0.99-1.37) |
|  |  |  |  |  |  |  |
| Russian Federation | 26269 (23161-29551) | 14.55 (13-16.27) | 49417 (43755-56371) | 21.46 (19.17-24.17) | 1.46  (1.29-1.63) | 0.88 (0.82-0.94) |
| Rwanda | 344 (303-394) | 12.03 (10.69-13.51) | 929 (817-1060) | 15.89 (14.12-17.91) | 1.16  (1.02-1.29) | 1.7 (1.54-1.86) |
| Saint Kitts and Nevis | 12 (10-15) | 33.67 (27.7-42) | 35 (28-44) | 47.65 (39.61-58.66) | 0.99  (0.78-1.2) | 1.83 (1.42-2.27) |
| Saint Lucia | 23 (20-26) | 25.68 (22.62-29.06) | 84 (75-96) | 38.74 (34.28-44.1) | 1.33  (1.19-1.47) | 2.69 (2.47-2.92) |
| Saint Vincent and the Grenadines | 17 (15-20) | 24.04 (20.9-27.11) | 48 (42-53) | 34.63 (30.59-38.74) | 1.22  (1.1-1.34) | 1.76 (1.59-1.93) |
| Samoa | 22 (19-25) | 24.53 (21.49-27.8) | 50 (44-56) | 33.82 (30.02-37.99) | 0.93  (0.82-1.04) | 1.25 (1.12-1.38) |
| San Marino | 8 (7-9) | 23.75 (20.95-26.82) | 17 (15-19) | 26.64 (23.47-30.16) | 0.36  (0.31-0.4) | 1.09 (0.96-1.22) |
| Sao Tome and Principe | 13 (12-15) | 20.27 (17.99-22.75) | 30 (27-35) | 29.24 (26-32.86) | 1.33  (1.27-1.39) | 1.25 (1.12-1.38) |
| Saudi Arabia | 2504 (2193-2850) | 42.25 (37.43-47.77) | 15220 (13267-17445) | 78.37 (70.88-86.26) | 2.09  (1.97-2.2) | 5.08 (4.7-5.53) |
| Senegal | 558 (491-632) | 17.26 (15.31-19.37) | 1614 (1426-1813) | 21.73 (19.35-24.18) | 0.82  (0.76-0.88) | 1.89 (1.76-2.05) |
| Serbia | 2277 (1991-2592) | 19.16 (16.92-21.55) | 5380 (4672-6122) | 32.71 (28.73-36.82) | 1.92  (1.86-1.98) | 1.36 (1.14-1.6) |
| Seychelles | 16 (14-18) | 28.56 (24.9-32.4) | 49 (43-55) | 43.11 (38.08-48.01) | 1.42  (1.3-1.53) | 2.02 (1.79-2.28) |
| Sierra Leone | 287 (252-324) | 14.97 (13.24-16.8) | 713 (625-809) | 20.29 (17.78-22.79) | 1.09  (1-1.17) | 1.48 (1.35-1.63) |
| Singapore | 753 (695-818) | 34.82 (32.18-37.53) | 3162 (2837-3523) | 39.98 (36.04-44.29) | 0.51  (0.45-0.56) | 3.2 (2.88-3.54) |
| Slovakia | 1228 (1081-1401) | 20.35 (17.96-23.09) | 2796 (2400-3206) | 29.77 (25.79-33.93) | 1.27  (1.22-1.31) | 1.28 (1.12-1.48) |
| Slovenia | 453 (396-515) | 18.52 (16.15-20.95) | 1196 (1047-1335) | 28.45 (24.96-31.77) | 1.58  (1.53-1.62) | 1.64 (1.42-1.88) |
| Solomon Islands | 27 (23-33) | 18.2 (15.79-20.94) | 69 (58-81) | 21.21 (18.37-24.3) | 0.38  (0.28-0.49) | 1.52 (1.36-1.68) |
| Somalia | 288 (254-329) | 12.11 (10.84-13.58) | 893 (782-1015) | 14.08 (12.6-15.72) | 0.58  (0.49-0.66) | 2.1 (1.95-2.27) |
| South Africa | 4501 (4002-5042) | 21.72 (19.39-24.3) | 13883 (12514-15494) | 30.89 (27.89-34.32) | 1.13  (0.95-1.31) | 2.08 (1.98-2.2) |
| South Sudan | 297 (261-333) | 12.74 (11.37-14.13) | 551 (486-621) | 15.05 (13.35-16.78) | 0.59  (0.54-0.64) | 0.85 (0.75-0.96) |
| Spain | 18008 (15974-20035) | 31.46 (28.04-34.82) | 29757 (26466-33032) | 30.62 (27.11-34.09) | -0.05  (-0.15-0.06) | 0.65 (0.54-0.79) |
| Sri Lanka | 2772 (2433-3139) | 25.15 (22.27-28.14) | 10449 (9105-11703) | 38.62 (33.86-42.91) | 1.51  (1.42-1.61) | 2.77 (2.5-3.06) |
| Sudan | 2614 (2291-2992) | 28.2 (24.88-31.86) | 9390 (8213-10629) | 52.03 (45.91-58.78) | 2.12  (2.08-2.17) | 2.59 (2.42-2.77) |
| Suriname | 55 (45-68) | 20.37 (16.85-25.18) | 238 (198-296) | 38.28 (32.05-47.24) | 2.26  (2.1-2.42) | 3.36 (2.97-3.78) |
| Sweden | 3567 (3096-4083) | 21.94 (19.24-24.79) | 5047 (4400-5794) | 23.11 (20.22-26.39) | 0.16  (0.15-0.18) | 0.41 (0.33-0.5) |
| Switzerland | 3245 (2860-3628) | 30.07 (26.59-33.39) | 5856 (5199-6506) | 33.15 (29.41-36.73) | 0.27  (0.2-0.34) | 0.8 (0.69-0.93) |
| Syrian Arab Republic | 1925 (1677-2207) | 36.82 (32.42-42.04) | 7753 (6909-8735) | 60.5 (54.46-67.77) | 1.89  (1.7-2.09) | 3.03 (2.7-3.33) |
| Taiwan (Province of China) | 5993 (5399-6615) | 36.98 (33.58-40.39) | 16915 (15650-18294) | 42.27 (39.15-45.54) | 0.4  (0.31-0.49) | 1.82 (1.64-2.04) |
| Tajikistan | 265 (221-317) | 9.22 (7.75-10.81) | 888 (737-1066) | 16.14 (13.69-18.88) | 2.12  (1.83-2.41) | 2.35 (2.1-2.6) |
| Thailand | 9763 (8530-11121) | 26.81 (23.62-30.21) | 42948 (38488-47554) | 41.01 (36.8-45.31) | 1.4  (1.28-1.53) | 3.4 (3.04-3.79) |
| Timor-Leste | 53 (46-61) | 18.13 (15.98-20.48) | 223 (193-253) | 26.38 (23.2-29.52) | 1.38  (1.29-1.47) | 3.2 (2.85-3.62) |
| Togo | 209 (183-238) | 17.16 (15.16-19.19) | 793 (695-898) | 22.02 (19.55-24.57) | 0.91  (0.82-1.01) | 2.78 (2.59-3) |
| Tokelau | 0 (0-0) | 19.81 (17.37-22.65) | 0 (0-0) | 31.69 (27.88-36.14) | 1.56  (1.54-1.59) | 0.6 (0.5-0.69) |
| Tonga | 14 (12-16) | 23.53 (20.65-26.76) | 26 (23-29) | 32.44 (28.62-36.41) | 0.97  (0.9-1.04) | 0.89 (0.79-0.99) |
| Trinidad and Tobago | 201 (176-228) | 23.46 (20.55-26.47) | 708 (622-797) | 37.07 (32.76-41.55) | 1.58  (1.49-1.67) | 2.52 (2.22-2.85) |
| Tunisia | 1834 (1600-2092) | 35.59 (31.37-40.36) | 8146 (7191-9163) | 63.23 (56.12-70.76) | 2.08  (1.93-2.23) | 3.44 (3.2-3.68) |
| Turkey | 10737 (9676-11988) | 30.82 (27.76-34.37) | 53090 (47509-59413) | 59.83 (53.44-66.76) | 2.58  (2.38-2.78) | 3.94 (3.51-4.39) |
| Turkmenistan | 266 (228-313) | 12.91 (11.23-14.82) | 836 (720-967) | 19.51 (17.06-22.08) | 1.54  (1.36-1.72) | 2.14 (1.89-2.47) |
| Tuvalu | 1 (1-2) | 19.05 (16.71-21.83) | 3 (3-4) | 30.19 (26.61-34.24) | 1.41  (1.33-1.48) | 1.34 (1.21-1.47) |
| Uganda | 717 (634-803) | 11.38 (10.16-12.68) | 1906 (1695-2153) | 14.3 (12.73-16.06) | 0.85  (0.76-0.94) | 1.66 (1.52-1.8) |
| Ukraine | 8122 (6953-9523) | 11.42 (9.82-13.32) | 10343 (9022-11775) | 13.88 (12.1-15.76) | 0.69  (0.47-0.9) | 0.27 (0.18-0.38) |
| United Arab Emirates | 219 (183-255) | 49.02 (43.27-54.9) | 3361 (2798-3960) | 75.43 (67.46-83.38) | 1.43  (1.29-1.57) | 14.37 (13.38-15.42) |
| United Kingdom | 25920 (22849-29289) | 26.91 (23.85-30.09) | 37559 (33252-41944) | 29.07 (25.82-32.48) | 0.02  (-0.11-0.15) | 0.45 (0.42-0.47) |
| United Republic of Tanzania | 1410 (1239-1584) | 13.08 (11.61-14.56) | 3933 (3475-4388) | 16.7 (14.87-18.56) | 0.83  (0.78-0.88) | 1.79 (1.63-1.95) |
| United States of America | 129418 (115102-144637) | 39.81 (35.66-44.36) | 241350 (217507-267987) | 42.95 (38.79-47.47) | 0.08  (-0.01-0.18) | 0.86 (0.79-0.94) |
| United States Virgin Islands | 22 (19-25) | 24.87 (22.04-27.71) | 72 (63-82) | 37.98 (33.57-42.55) | 1.42  (1.25-1.6) | 2.26 (2.02-2.53) |
| Uruguay | 929 (826-1045) | 22.9 (20.49-25.62) | 1656 (1498-1842) | 30.07 (27.28-33.49) | 0.92  (0.87-0.96) | 0.78 (0.64-0.95) |
| Uzbekistan | 1944 (1645-2280) | 16.31 (13.98-18.93) | 6081 (5064-7214) | 24.81 (21.24-28.74) | 1.48  (1.33-1.62) | 2.13 (1.94-2.33) |
| Vanuatu | 12 (10-14) | 17.81 (15.56-20.37) | 46 (40-53) | 25.8 (22.57-29.43) | 1.08  (0.98-1.18) | 2.76 (2.57-2.97) |
| Venezuela (Bolivarian Republic of) | 3272 (2841-3755) | 32.7 (28.69-37.43) | 14690 (13043-16407) | 48.81 (43.67-54.23) | 1.35  (1.25-1.46) | 3.49 (3.07-3.96) |
| Viet Nam | 6740 (5889-7677) | 16.64 (14.7-18.76) | 24355 (21277-27893) | 26.28 (23.16-29.55) | 1.71  (1.64-1.78) | 2.61 (2.37-2.91) |
| Yemen | 1322 (1149-1524) | 27.27 (23.95-31.09) | 6074 (5244-7033) | 46.96 (41.11-54.21) | 2.05  (2.01-2.09) | 3.59 (3.28-4.03) |
| Zambia | 390 (347-435) | 13.98 (12.55-15.47) | 1162 (1019-1296) | 18.12 (16.12-20.13) | 0.93  (0.83-1.04) | 1.98 (1.82-2.15) |
| Zimbabwe | 872 (759-1014) | 21.15 (18.63-24.11) | 1724 (1507-1995) | 24.73 (21.96-28.15) | 0.3  (0.01-0.6) | 0.98 (0.89-1.06) |
