# Supplementary material for: Epidemiological patterns of chronic kidney disease attributed to type 2 diabetes from 1990-2019
Source: Front Endocrinol (Lausanne). 2024 Apr 17;15:1383777. doi: 10.3389/fendo.2024.1383777 (PMC11061475; doi:10.3389/fendo.2024.1383777)
Supplement: Supplementary file 6 [file Table_2.docx]

**Supplementary Table 2.** DALYs of Chronic kidney disease due to diabetes mellitus type 2 in 1990 and 2019 for both sexes and all nations.

| Location | Numbers (95%UI)_1990 | Age-standardized rate (95%UI)_1990 | Numbers (95%UI)_2019 | Age-standardized rate (95%UI)_2019 | Estimated annual percentage changes (95%CI) | Numbers_change (95%UI) |
| --- | --- | --- | --- | --- | --- | --- |
| Afghanistan | 28085 (19306-40575) | 388.72 (269.17-566.22) | 43545 (30559-61669) | 351.65 (249.82-512.93) | -0.25  (-0.3--0.2) | 0.55 (0.09-1.1) |
| Albania | 822 (601-1066) | 38.95 (28.42-50.57) | 1437 (1045-1980) | 33.71 (24.75-45.99) | -0.74  (-0.96--0.52) | 0.75 (0.38-1.15) |
| Algeria | 24803 (17526-35990) | 218.13 (158.06-310.66) | 59538 (43839-81257) | 187.54 (138.83-254.08) | -0.38  (-0.54--0.21) | 1.4 (0.79-2.18) |
| American Samoa | 77 (61-97) | 324.64 (261.79-400.63) | 233 (182-291) | 478.35 (379.02-589.82) | 1.41  (1.27-1.54) | 2.01 (1.48-2.65) |
| Andorra | 19 (14-27) | 38.57 (28.09-53.18) | 50 (36-67) | 34.08 (24.74-46.33) | -0.43  (-0.56--0.3) | 1.59 (0.88-2.35) |
| Angola | 5228 (3488-7242) | 135.93 (91.28-181.22) | 13808 (9210-19375) | 128.66 (86.42-175.66) | -0.27  (-0.31--0.22) | 1.64 (0.98-2.6) |
| Antigua and Barbuda | 93 (75-111) | 178.32 (142.46-215.83) | 252 (196-311) | 247.76 (194.52-304.71) | 1.37  (1.2-1.54) | 1.71 (1.29-2.21) |
| Argentina | 45878 (35903-55324) | 142.57 (112.64-171.54) | 87630 (68843-106326) | 161.92 (126.91-196.85) | 0.37  (0.09-0.65) | 0.91 (0.72-1.12) |
| Armenia | 683 (547-830) | 25.7 (20.77-30.89) | 3563 (2776-4422) | 86.64 (67.88-107.23) | 4.26  (3.95-4.58) | 4.22 (3.35-5.29) |
| Australia | 2990 (2337-3816) | 15.55 (12.29-19.62) | 10070 (7531-13085) | 23.43 (17.6-30.15) | 1.71  (1.49-1.93) | 2.37 (1.76-3.13) |
| Austria | 3615 (2764-4585) | 30.06 (23.09-37.99) | 10712 (8237-13886) | 54.35 (41.88-69.23) | 2.9  (2.37-3.42) | 1.96 (1.56-2.42) |
| Azerbaijan | 4028 (3083-5008) | 74.76 (58.18-92.26) | 12683 (9771-15908) | 131.85 (103.62-164.83) | 2.44  (2.04-2.84) | 2.15 (1.59-2.85) |
| Bahamas | 270 (214-326) | 169.43 (134.72-203.34) | 821 (622-1039) | 204.93 (157.85-256.19) | 0.95  (0.82-1.07) | 2.04 (1.52-2.74) |
| Bahrain | 382 (296-475) | 246.93 (193.56-311.21) | 1776 (1345-2239) | 219.49 (171.24-276.69) | -0.38  (-0.85-0.1) | 3.65 (2.61-4.9) |
| Bangladesh | 36630 (26357-48402) | 79.07 (57.55-103.54) | 92073 (67201-122066) | 71.09 (52.82-93.88) | 0.04  (-0.21-0.29) | 1.51 (0.93-2.32) |
| Barbados | 356 (293-419) | 127.15 (103.68-150.63) | 845 (665-1045) | 174.4 (137.81-214.82) | 1.07  (0.89-1.25) | 1.38 (1.01-1.79) |
| Belarus | 1437 (1092-1846) | 11.29 (8.62-14.58) | 2308 (1717-3047) | 14.73 (10.91-19.4) | 1.13  (0.93-1.34) | 0.61 (0.39-0.86) |
| Belgium | 5998 (4575-7711) | 38.3 (29.17-48.49) | 8579 (6429-11361) | 34.64 (26.15-44.94) | -0.23  (-0.32--0.15) | 0.43 (0.25-0.64) |
| Belize | 139 (111-168) | 148.92 (119.09-179.24) | 794 (632-979) | 275.37 (220.29-336.85) | 2.3  (1.86-2.75) | 4.7 (3.84-5.73) |
| Benin | 3589 (2645-4609) | 181.96 (133.8-232.62) | 8548 (6037-11777) | 177.16 (128.55-239.71) | 0.05  (-0.04-0.14) | 1.38 (0.79-2.15) |
| Bermuda | 70 (56-84) | 111.66 (89.2-134.19) | 128 (103-159) | 101.47 (81.14-125.74) | -0.13  (-0.25--0.01) | 0.84 (0.55-1.2) |
| Bhutan | 351 (214-515) | 138 (86.35-201.52) | 1013 (700-1374) | 181.01 (126.02-244.44) | 1.05  (0.99-1.12) | 1.89 (1.06-3.12) |
| Bolivia (Plurinational State of) | 7291 (5455-9566) | 229.27 (172.47-298.05) | 27113 (19636-36236) | 314.93 (228.62-418.68) | 1.16  (1.09-1.23) | 2.72 (1.84-3.84) |
| Bosnia and Herzegovina | 1804 (1306-2325) | 43.37 (31.85-55.51) | 3553 (2559-4754) | 58.99 (43.36-78.3) | 0.92  (0.34-1.5) | 0.97 (0.57-1.49) |
| Botswana | 764 (494-1108) | 134.17 (87.75-192.06) | 2764 (1801-4011) | 202.13 (136.12-289.66) | 0.95  (0.52-1.39) | 2.62 (1.54-4.04) |
| Brazil | 96297 (77500-114873) | 106.69 (86.65-126.43) | 245480 (199781-291254) | 103.75 (84.78-122.4) | -0.23  (-0.35--0.11) | 1.55 (1.36-1.74) |
| Brunei Darussalam | 313 (255-376) | 332.17 (278.24-390.21) | 850 (688-1009) | 306.14 (255.06-357.56) | 0.28  (0.03-0.54) | 1.72 (1.26-2.25) |
| Bulgaria | 5185 (3821-6602) | 41.3 (31.05-51.95) | 10709 (7661-14497) | 78.19 (55.71-107.3) | 2.26  (1.99-2.54) | 1.07 (0.71-1.5) |
| Burkina Faso | 6588 (4747-8608) | 157.41 (116.76-202.06) | 14806 (10959-19652) | 167.16 (125.84-219.5) | 0.14  (0.01-0.26) | 1.25 (0.76-1.88) |
| Burundi | 4122 (2953-5606) | 178.04 (128-239.75) | 5893 (4153-7918) | 135.97 (96.9-178.61) | -1.25  (-1.37--1.13) | 0.43 (0.08-0.93) |
| Cabo Verde | 187 (145-236) | 80.94 (62.14-101.62) | 574 (446-712) | 135.12 (105.65-166.46) | 1.07  (0.8-1.35) | 2.07 (1.44-2.71) |
| Cambodia | 10974 (8129-14210) | 214.2 (163.5-272.87) | 22792 (17324-29341) | 181.11 (140.56-228.34) | -0.69  (-0.78--0.6) | 1.08 (0.56-1.78) |
| Cameroon | 11802 (8309-15798) | 269.83 (194.41-356.43) | 31105 (21794-43108) | 261.38 (190.13-352.84) | -0.1  (-0.22-0.02) | 1.64 (0.95-2.63) |
| Canada | 13282 (10181-16564) | 40.73 (31.44-50.58) | 28433 (21818-36012) | 40.65 (31.08-51.4) | -0.21  (-0.33--0.09) | 1.14 (0.92-1.38) |
| Central African Republic | 2047 (1394-2790) | 173.47 (119.53-233.77) | 3835 (2520-5481) | 175.5 (118.28-244.46) | 0.07  (-0.06-0.2) | 0.87 (0.39-1.52) |
| Chad | 4591 (3147-6701) | 164.17 (112.54-238.43) | 9655 (6585-13638) | 173.66 (121.88-243.71) | 0.31  (0.2-0.42) | 1.1 (0.61-1.71) |
| Chile | 8457 (6460-10279) | 86.42 (66.14-105.06) | 27007 (21045-33286) | 111.99 (87.71-137.79) | 1.08  (0.72-1.44) | 2.19 (1.87-2.57) |
| China | 849044 (663737-1032537) | 97.41 (78.35-116.36) | 1640410 (1312349-1979868) | 82.96 (66.87-99) | -0.07  (-0.23-0.09) | 0.93 (0.6-1.34) |
| Colombia | 22995 (18307-27517) | 130.75 (105.14-156.17) | 61776 (47134-80005) | 116.51 (88.86-151.77) | -0.58  (-0.73--0.42) | 1.69 (1.17-2.32) |
| Comoros | 346 (204-474) | 159.92 (99.94-218.81) | 681 (503-898) | 144.63 (108.1-189.57) | -0.55  (-0.69--0.41) | 0.97 (0.47-2.13) |
| Congo | 2174 (1413-3094) | 206.17 (135.2-286.49) | 4314 (2826-6098) | 171.39 (113.14-236.88) | -0.83  (-0.95--0.71) | 0.98 (0.47-1.63) |
| Cook Islands | 27 (21-33) | 204.79 (163.29-249.15) | 62 (50-77) | 254.58 (207-316.3) | 0.9  (0.83-0.98) | 1.33 (0.86-1.92) |
| Costa Rica | 2137 (1721-2581) | 122.38 (98.58-148.24) | 9599 (7241-12404) | 185.26 (140.6-239.83) | 1.23  (0.72-1.75) | 3.49 (2.7-4.49) |
| Côte d'Ivoire | 7973 (5474-10738) | 201.25 (145.65-263.86) | 19255 (13429-26057) | 183.3 (131.14-242.56) | -0.51  (-0.69--0.32) | 1.42 (0.8-2.22) |
| Croatia | 2980 (2235-3760) | 46.37 (34.98-58.11) | 4716 (3499-6167) | 52.98 (39.66-68.36) | 0.23  (-0.06-0.52) | 0.58 (0.3-0.93) |
| Cuba | 6120 (4931-7335) | 59.41 (47.84-71.38) | 18735 (14239-24126) | 100.6 (76.38-129.34) | 2.18  (1.98-2.38) | 2.06 (1.56-2.68) |
| Cyprus | 876 (635-1178) | 123.34 (91.87-165.18) | 1331 (1007-1778) | 71.33 (54.38-93.62) | -2.22  (-2.33--2.1) | 0.52 (0.24-0.82) |
| Czechia | 6887 (5170-8684) | 50.25 (37.84-63.39) | 7794 (5813-10010) | 37.41 (28.24-47.65) | -0.99  (-1.07--0.91) | 0.13 (-0.05-0.35) |
| Democratic People's Republic of Korea | 23973 (17697-30922) | 140.84 (107.43-178.4) | 43649 (33946-55201) | 134.34 (105.06-169.19) | -0.13  (-0.21--0.05) | 0.82 (0.43-1.34) |
| Democratic Republic of the Congo | 24106 (17410-31742) | 158.53 (119-203.01) | 45926 (32636-61998) | 132.72 (94.38-178.38) | -0.71  (-0.76--0.66) | 0.91 (0.43-1.51) |
| Denmark | 2072 (1574-2646) | 25.48 (19.29-32.16) | 4460 (3406-5754) | 37.28 (28.91-47.81) | 1  (0.72-1.28) | 1.15 (0.91-1.45) |
| Djibouti | 169 (112-242) | 127.53 (87.54-178.98) | 872 (601-1197) | 158.19 (113.97-212.8) | 0.74  (0.65-0.84) | 4.17 (2.71-6.16) |
| Dominica | 161 (131-193) | 231.33 (186.67-279.95) | 270 (210-342) | 302.22 (235.01-380.87) | 1.15  (1.06-1.23) | 0.68 (0.36-1.06) |
| Dominican Republic | 3393 (2635-4163) | 88.47 (69.46-108.49) | 14477 (10302-20145) | 153.78 (109.91-211.73) | 2.72  (2.44-2.99) | 3.27 (2.25-4.53) |
| Ecuador | 6197 (4914-7433) | 116.24 (92.83-139.1) | 40339 (29770-52921) | 272.56 (203.76-356.78) | 3.23  (2.61-3.85) | 5.51 (4.25-7.23) |
| Egypt | 68875 (45839-90852) | 246.45 (167.6-322.24) | 184235 (109809-273243) | 302.75 (184.53-442.24) | 0.89  (0.81-0.96) | 1.67 (0.91-2.47) |
| El Salvador | 4509 (3557-5461) | 149.27 (118.06-180.61) | 27510 (19979-36917) | 470.92 (342.22-632.72) | 4.32  (3.6-5.04) | 5.1 (3.65-6.91) |
| Equatorial Guinea | 320 (211-440) | 161.72 (109.66-220.08) | 788 (524-1166) | 173.89 (119.95-249.64) | 0.43  (0.27-0.58) | 1.47 (0.68-2.7) |
| Eritrea | 1463 (941-2133) | 146.96 (92.85-213.68) | 4090 (2710-5831) | 159.24 (107.96-223.44) | 0.19  (0.05-0.32) | 1.8 (1.04-2.89) |
| Estonia | 314 (236-400) | 15.51 (11.7-19.81) | 1160 (838-1575) | 41.74 (30.37-56.05) | 3.65  (3.26-4.05) | 2.7 (1.96-3.63) |
| Eswatini | 596 (416-800) | 203.66 (144.75-269.68) | 1755 (1162-2463) | 303.05 (204.87-409.81) | 1.57  (0.93-2.22) | 1.94 (1.13-3.16) |
| Ethiopia | 46149 (34304-58649) | 232.59 (175.89-291.49) | 55829 (44066-68961) | 142.92 (113.27-175.2) | -1.74  (-1.86--1.63) | 0.21 (-0.04-0.51) |
| Fiji | 1202 (899-1567) | 305.2 (235.41-393.33) | 2978 (2260-3857) | 392.55 (308.19-500.62) | 0.4  (0.06-0.74) | 1.48 (0.85-2.35) |
| Finland | 1514 (1199-1871) | 21.14 (16.76-25.87) | 3277 (2571-4100) | 25.15 (19.8-31.27) | 1.12  (0.91-1.33) | 1.16 (0.9-1.48) |
| France | 24090 (18359-30857) | 28.02 (21.46-35.41) | 37488 (28346-48687) | 25.05 (19.2-32.29) | -0.56  (-0.68--0.44) | 0.56 (0.36-0.76) |
| Gabon | 1097 (747-1489) | 199.61 (136.15-272.49) | 2234 (1343-3140) | 224.29 (135.38-312.57) | 0.36  (0.13-0.58) | 1.04 (0.47-1.7) |
| Gambia | 545 (375-750) | 158.65 (113.11-214.74) | 1662 (1193-2246) | 175.54 (128.15-233.27) | 0.28  (0.15-0.42) | 2.05 (1.16-3.23) |
| Georgia | 3173 (2444-3947) | 51.26 (39.75-63.46) | 5649 (4336-7086) | 99.61 (76.55-125.21) | 3.73  (3.04-4.42) | 0.78 (0.47-1.16) |
| Germany | 58385 (45600-73229) | 45.22 (35.14-56.58) | 131186 (100604-171012) | 61.25 (47.37-78.6) | 1.39  (1.16-1.62) | 1.25 (0.9-1.62) |
| Ghana | 9701 (6660-13541) | 156.16 (110.91-213.08) | 30058 (20494-41949) | 190.15 (133.43-260.78) | 0.91  (0.74-1.08) | 2.1 (1.1-3.17) |
| Greece | 12540 (9427-16210) | 81.64 (62.09-104.7) | 15590 (11847-20338) | 60.83 (46.97-77.57) | -0.55  (-0.86--0.23) | 0.24 (0.09-0.4) |
| Greenland | 22 (17-28) | 70.35 (53.58-88.28) | 39 (28-52) | 58.76 (43.63-77.44) | -0.78  (-0.94--0.63) | 0.78 (0.42-1.16) |
| Grenada | 160 (129-193) | 230.3 (184.53-277.62) | 365 (291-447) | 322.01 (259.52-390.56) | 1.53  (1.39-1.67) | 1.28 (0.97-1.62) |
| Guam | 148 (118-177) | 190.9 (156.17-228.05) | 505 (396-625) | 263.58 (205.38-324.98) | 1.38  (1.18-1.59) | 2.42 (1.81-3.15) |
| Guatemala | 7098 (5540-8820) | 194.3 (154.47-239.21) | 42110 (31666-54731) | 381.61 (288.78-496.39) | 3.13  (2.72-3.54) | 4.93 (3.69-6.51) |
| Guinea | 6002 (4108-8213) | 183.46 (126.36-252.84) | 10125 (7180-13809) | 184.85 (133.24-245.92) | -0.03  (-0.12-0.07) | 0.69 (0.26-1.23) |
| Guinea-Bissau | 1199 (836-1640) | 291.54 (207.39-387.78) | 1797 (1261-2440) | 242.39 (176.61-325.24) | -0.58  (-0.63--0.52) | 0.5 (0.1-1.03) |
| Guyana | 775 (607-971) | 196.93 (156.85-243.05) | 2173 (1578-2893) | 330.42 (244.88-434.74) | 2.47  (2.25-2.7) | 1.8 (1.17-2.62) |
| Haiti | 7610 (5303-11203) | 222.73 (157.51-330.41) | 16384 (10898-25611) | 224.8 (152.72-346.33) | 0.4  (0.24-0.55) | 1.15 (0.65-1.82) |
| Honduras | 3731 (2724-5248) | 175.44 (126.64-248.86) | 23159 (17086-30685) | 381.08 (282.47-504.96) | 3.09  (2.84-3.35) | 5.21 (3.9-6.87) |
| Hungary | 5235 (3977-6462) | 35.88 (27.81-43.95) | 9260 (7016-11950) | 47.75 (36.72-61.53) | 1.97  (1.32-2.62) | 0.77 (0.49-1.09) |
| Iceland | 49 (38-63) | 17.04 (13.21-21.78) | 104 (79-136) | 18.13 (13.81-23.8) | 0  (-0.43-0.44) | 1.1 (0.86-1.37) |
| India | 601245 (446101-773108) | 130.69 (99.63-163.89) | 1648772 (1227573-2117240) | 142.09 (107.11-180.96) | 0.33  (0.07-0.58) | 1.74 (1.28-2.32) |
| Indonesia | 203041 (157198-249577) | 178.25 (141.51-214.97) | 428026 (329027-539663) | 183.2 (144.46-225.82) | 0.11  (0.02-0.2) | 1.11 (0.74-1.55) |
| Iran (Islamic Republic of) | 37969 (30416-44855) | 152.72 (124.83-180.36) | 89014 (73930-103507) | 126.85 (105.48-147.59) | -0.81  (-0.92--0.7) | 1.34 (1.09-1.6) |
| Iraq | 25105 (18999-32801) | 327.39 (249.41-430.37) | 65574 (48920-86996) | 297.51 (225.49-383.86) | -0.43  (-0.48--0.39) | 1.61 (0.97-2.36) |
| Ireland | 1855 (1398-2404) | 44.87 (34.45-57.51) | 2655 (2040-3422) | 34.78 (26.91-44.46) | -1.32  (-1.52--1.12) | 0.43 (0.25-0.63) |
| Israel | 4605 (3542-5803) | 95.7 (74.04-119.95) | 11776 (9141-14702) | 98.15 (75.85-122.92) | 0.47  (-0.29-1.24) | 1.56 (1.27-1.87) |
| Italy | 34684 (26798-43775) | 38.52 (29.78-47.92) | 47246 (36367-60634) | 29.7 (23.05-37.5) | -1.07  (-1.39--0.75) | 0.36 (0.21-0.53) |
| Jamaica | 2621 (2138-3097) | 147.8 (120.21-174.93) | 6507 (4992-8370) | 217.29 (166.61-278.39) | 0.88  (0.42-1.34) | 1.48 (0.99-2.07) |
| Japan | 157737 (131523-182375) | 94.63 (79.33-109.41) | 257021 (208187-304582) | 69.71 (56.93-83.23) | -0.88  (-1.1--0.65) | 0.63 (0.45-0.8) |
| Jordan | 3577 (2813-4455) | 284.26 (225.17-351.51) | 15674 (12281-19450) | 255.54 (200.25-316.67) | -0.31  (-0.48--0.13) | 3.38 (2.5-4.49) |
| Kazakhstan | 8085 (6279-10000) | 59.71 (46.86-72.99) | 16631 (12878-20819) | 93.39 (72.59-115.43) | 1.08  (0.8-1.37) | 1.06 (0.74-1.41) |
| Kenya | 8003 (5974-10461) | 102.13 (77.04-133.15) | 26204 (19590-33312) | 126.82 (96.39-160.25) | 0.84  (0.73-0.94) | 2.27 (1.72-2.86) |
| Kiribati | 170 (127-221) | 409.07 (310.95-523.01) | 386 (266-529) | 502.12 (360.62-677.42) | 0.42  (0.04-0.81) | 1.27 (0.64-2.02) |
| Kuwait | 1103 (873-1335) | 186.92 (149.12-223.33) | 2783 (2204-3464) | 116.07 (90.5-144.05) | -1.89  (-2.57--1.21) | 1.52 (1.12-2.07) |
| Kyrgyzstan | 3069 (2308-3907) | 95.35 (71.36-120.6) | 4103 (3094-5343) | 78.35 (59.56-100.3) | -1.28  (-1.68--0.88) | 0.34 (0.11-0.61) |
| Lao People's Democratic Republic | 10624 (7584-14033) | 471.49 (340.12-613.83) | 17714 (12868-23881) | 380.41 (283.82-499.12) | -0.91  (-0.98--0.85) | 0.67 (0.23-1.25) |
| Latvia | 397 (297-526) | 11.28 (8.4-14.99) | 886 (655-1167) | 22.67 (16.59-29.92) | 3.12  (2.8-3.44) | 1.23 (0.89-1.65) |
| Lebanon | 4579 (3506-5882) | 207.17 (159.42-263.14) | 7995 (5871-10743) | 153.95 (113.18-206.14) | -0.95  (-1.13--0.78) | 0.75 (0.29-1.21) |
| Lesotho | 1205 (829-1684) | 122.89 (86.04-169.38) | 3484 (2312-5038) | 275.77 (187.35-387.53) | 3.43  (3.09-3.77) | 1.89 (1.08-2.97) |
| Liberia | 2358 (1687-3115) | 216.52 (156.14-284.63) | 3677 (2467-5282) | 180.9 (124.79-252.37) | -0.4  (-0.69--0.11) | 0.56 (0.12-1.11) |
| Libya | 3601 (2570-4726) | 197.77 (141.5-260.39) | 10521 (7113-14249) | 212.41 (143.31-287.49) | 0.41  (0.27-0.55) | 1.92 (1.18-2.89) |
| Lithuania | 573 (429-744) | 12.8 (9.55-16.67) | 1002 (758-1295) | 18.06 (13.47-23.7) | 1.03  (0.75-1.31) | 0.75 (0.51-1.03) |
| Luxembourg | 228 (175-293) | 41.62 (32.01-53.21) | 423 (324-561) | 40.4 (30.75-53.01) | 0.08  (-0.13-0.29) | 0.85 (0.62-1.14) |
| Madagascar | 5979 (4265-8043) | 118.65 (83.78-158.9) | 11672 (8312-16533) | 112.12 (79.98-154.3) | -0.3  (-0.39--0.21) | 0.95 (0.43-1.58) |
| Malawi | 5348 (3969-6786) | 144.84 (109.59-183.66) | 9948 (7375-12974) | 144.2 (108.24-184.09) | -0.18  (-0.35--0.01) | 0.86 (0.46-1.35) |
| Malaysia | 22240 (18066-26357) | 231.54 (189.39-270.8) | 63695 (49720-80374) | 235.26 (186.27-293.81) | -0.38  (-0.57--0.18) | 1.86 (1.3-2.56) |
| Maldives | 443 (345-562) | 474.13 (381.23-593.36) | 784 (623-974) | 259.69 (206.86-321.16) | -2.49  (-2.78--2.2) | 0.77 (0.38-1.24) |
| Mali | 7902 (5553-10720) | 193.29 (138.41-256.56) | 14402 (10209-19254) | 170.07 (122.67-223.97) | -0.28  (-0.46--0.11) | 0.82 (0.39-1.34) |
| Malta | 239 (182-308) | 56.94 (43.68-72.72) | 424 (320-550) | 44.16 (33.64-56.8) | -0.85  (-1.05--0.66) | 0.78 (0.56-1.04) |
| Marshall Islands | 60 (45-80) | 338.34 (255.45-450.29) | 172 (117-245) | 449.18 (318.16-619.34) | 0.96  (0.72-1.19) | 1.85 (1.18-2.65) |
| Mauritania | 2589 (1862-3418) | 260.84 (190.41-338.2) | 3416 (2340-4623) | 168.77 (118.37-224.59) | -1.43  (-1.53--1.32) | 0.32 (0-0.69) |
| Mauritius | 3274 (2683-3856) | 427.98 (352.92-498.37) | 13104 (10204-16731) | 733.03 (578.1-932.24) | 2.2  (1.83-2.57) | 3 (2.23-3.94) |
| Mexico | 80036 (65580-94199) | 186.6 (155.67-217.85) | 487385 (383517-604961) | 410.66 (324.66-508.17) | 3.01  (2.56-3.46) | 5.09 (4.34-5.93) |
| Micronesia (Federated States of) | 212 (156-284) | 425.29 (317.65-562.28) | 502 (345-702) | 657.71 (471.95-897.48) | 1.41  (1.05-1.77) | 1.37 (0.6-2.35) |
| Monaco | 16 (12-21) | 22.5 (17.15-29.21) | 28 (21-37) | 27.92 (20.82-36.22) | 0.95  (0.69-1.21) | 0.72 (0.45-1.05) |
| Mongolia | 2431 (1772-3186) | 219.9 (161.83-288.75) | 3220 (2253-4407) | 121.95 (88.03-162.2) | -3.18  (-3.62--2.74) | 0.32 (0.01-0.77) |
| Montenegro | 419 (318-524) | 67.2 (51.22-83.95) | 767 (561-997) | 77.7 (57.71-100.76) | 0.29  (0.14-0.43) | 0.83 (0.49-1.2) |
| Morocco | 23987 (17784-31803) | 179.3 (133.16-243.07) | 69257 (50004-90704) | 228.48 (166.7-298.92) | 1.17  (1.04-1.3) | 1.89 (1.14-2.64) |
| Mozambique | 6715 (4698-9055) | 118.26 (83.72-157.83) | 14665 (10437-19988) | 140.26 (101.71-186.62) | 0.62  (0.5-0.75) | 1.18 (0.52-1.95) |
| Myanmar | 71721 (50935-94922) | 276.43 (201.77-364.2) | 115696 (88671-151033) | 239.14 (188.72-306.73) | -0.58  (-0.69--0.48) | 0.61 (0.21-1.18) |
| Namibia | 935 (606-1403) | 131.48 (86.66-196.16) | 1813 (1204-2632) | 131.79 (88.76-188.25) | -0.4  (-0.81-0) | 0.94 (0.42-1.57) |
| Nauru | 18 (13-25) | 416.54 (304.6-548.72) | 28 (19-39) | 558.49 (399.25-735.3) | 0.9  (0.57-1.22) | 0.53 (0.19-0.92) |
| Nepal | 10716 (7040-15054) | 109.35 (72.95-152.14) | 36904 (24907-50800) | 164 (111.37-224.08) | 1.48  (1.19-1.77) | 2.44 (1.55-3.51) |
| Netherlands | 5805 (4400-7489) | 28.65 (21.71-36.48) | 11057 (8444-14449) | 30.83 (23.54-39.97) | 0.47  (0.28-0.67) | 0.9 (0.72-1.1) |
| New Zealand | 1015 (769-1319) | 26.36 (20.11-33.98) | 2682 (2028-3443) | 34.5 (26.3-44.19) | 0.96  (0.61-1.31) | 1.64 (1.37-1.94) |
| Nicaragua | 4015 (3135-4913) | 254.42 (201.29-308.96) | 24805 (18307-31940) | 552.02 (412.19-706.28) | 3.05  (2.76-3.34) | 5.18 (4.05-6.46) |
| Niger | 4965 (3431-6839) | 180.99 (127.49-246.63) | 11681 (8127-16081) | 153.47 (110.13-207.24) | -0.49  (-0.58--0.39) | 1.35 (0.84-2.09) |
| Nigeria | 53923 (37425-74688) | 128.71 (90.55-176.02) | 97878 (70339-131348) | 118.66 (86.37-156.84) | -0.51  (-0.68--0.33) | 0.82 (0.38-1.37) |
| Niue | 7 (5-8) | 303.95 (231.77-389.26) | 9 (6-11) | 401.23 (292.63-519.83) | 0.82  (0.58-1.06) | 0.33 (0.05-0.67) |
| North Macedonia | 1070 (782-1379) | 55.82 (41.46-71.88) | 2231 (1631-2962) | 68.6 (50.5-90.72) | 0.58  (0.34-0.82) | 1.08 (0.7-1.57) |
| Northern Mariana Islands | 76 (56-101) | 367.36 (290.7-459.73) | 246 (187-308) | 449 (354.95-553.99) | 0.86  (0.76-0.96) | 2.24 (1.47-3.14) |
| Norway | 1516 (1158-1962) | 21.58 (16.59-27.61) | 2516 (1944-3196) | 24.72 (19.27-31.24) | 0.67  (0.5-0.83) | 0.66 (0.51-0.84) |
| Oman | 724 (536-968) | 120.16 (89.96-159.98) | 2058 (1658-2497) | 142.42 (115.43-171.52) | 0.86  (0.72-0.99) | 1.84 (1.19-2.64) |
| Pakistan | 85312 (58016-120486) | 147.9 (101.14-209.16) | 274446 (192011-372463) | 235.83 (169.06-317.3) | 1.61  (1.34-1.87) | 2.22 (1.47-3.05) |
| Palau | 47 (36-63) | 458.54 (351.89-601.14) | 129 (95-172) | 580.37 (444.47-746.57) | 0.75  (0.54-0.95) | 1.74 (1-2.62) |
| Palestine | 2581 (1895-3401) | 303.12 (224.58-402.17) | 5257 (4155-6472) | 233.18 (186.39-286.11) | -0.62  (-0.88--0.35) | 1.04 (0.56-1.64) |
| Panama | 1607 (1289-1919) | 106.44 (85.64-126.67) | 7807 (5882-10124) | 188.22 (141.42-244.04) | 1.99  (1.68-2.3) | 3.86 (2.88-4.96) |
| Papua New Guinea | 2441 (1801-3150) | 112.19 (85.51-141.95) | 7556 (5479-10318) | 133.46 (100.65-175.9) | 0.52  (0.41-0.64) | 2.1 (1.43-2.98) |
| Paraguay | 2195 (1738-2645) | 99.13 (79.01-119.21) | 12815 (9335-16979) | 230.5 (169.86-302.18) | 3.52  (3.29-3.74) | 4.84 (3.49-6.56) |
| Peru | 14818 (11224-18719) | 124.96 (94.88-156.74) | 42778 (30999-57904) | 133.95 (97.28-180.91) | 0.33  (0.14-0.51) | 1.89 (1.18-2.84) |
| Philippines | 89876 (70868-109380) | 285.51 (232.67-341.66) | 319626 (242430-404655) | 382.99 (294.27-478.45) | 1.41  (1.15-1.68) | 2.56 (1.84-3.38) |
| Poland | 26288 (20314-32297) | 60.07 (46.77-73.14) | 26990 (20773-33793) | 38.91 (30.09-48.52) | -1.26  (-1.67--0.85) | 0.03 (-0.12-0.19) |
| Portugal | 7215 (5375-9287) | 51.95 (39.09-65.78) | 13021 (9853-16795) | 49.51 (37.64-63.09) | 0.11  (-0.42-0.64) | 0.8 (0.53-1.11) |
| Puerto Rico | 6659 (5430-7749) | 184.77 (151.29-215.58) | 13110 (10085-16768) | 192.57 (146.94-246.5) | 0.63  (0.42-0.83) | 0.97 (0.6-1.38) |
| Qatar | 255 (193-370) | 303.37 (228.43-485.64) | 1528 (1161-1981) | 256.95 (199.91-333.83) | -0.34  (-0.65--0.03) | 5 (3.32-7.03) |
| Republic of Korea | 32847 (27693-37323) | 109.79 (94.08-123.8) | 74259 (64329-84423) | 83.44 (72.36-94.89) | -0.79  (-0.92--0.66) | 1.26 (0.99-1.57) |
| Republic of Moldova | 521 (395-670) | 12.3 (9.49-15.67) | 1119 (824-1459) | 19.37 (14.4-24.98) | 1.67  (1.41-1.94) | 1.15 (0.88-1.46) |
| Romania | 8119 (5470-10594) | 29.06 (19.74-37.29) | 14652 (10800-19072) | 40.45 (30.04-52.57) | 1.7  (1.32-2.09) | 0.8 (0.48-1.52) |
| Russian Federation | 39637 (29946-51487) | 22.04 (16.76-28.21) | 55780 (42329-72032) | 23.85 (18.18-30.47) | 0.09  (-0.08-0.27) | 0.41 (0.27-0.56) |
| Rwanda | 5304 (3886-6912) | 185.72 (138.66-239.49) | 7573 (5475-10062) | 134.63 (99.3-175.77) | -1.92  (-2.25--1.58) | 0.43 (0.11-0.84) |
| Saint Kitts and Nevis | 111 (89-135) | 308.46 (245.7-374.46) | 237 (179-305) | 349.09 (271.83-436.62) | 0.58  (0.44-0.73) | 1.13 (0.71-1.63) |
| Saint Lucia | 179 (144-213) | 206.07 (165.87-244.39) | 488 (381-606) | 225.56 (176.78-280.08) | 0.41  (0.22-0.6) | 1.73 (1.28-2.23) |
| Saint Vincent and the Grenadines | 122 (98-147) | 170.92 (137.07-206.08) | 332 (264-407) | 245.63 (195.68-300.45) | 1.44  (1.27-1.61) | 1.72 (1.33-2.17) |
| Samoa | 288 (212-387) | 319.3 (238.58-427.82) | 569 (427-736) | 377.18 (290.94-480.31) | 0.49  (0.29-0.68) | 0.98 (0.49-1.68) |
| San Marino | 7 (5-9) | 20.63 (15.84-27.18) | 16 (11-22) | 23.13 (16.46-31.74) | 0.74  (0.61-0.87) | 1.32 (0.79-1.98) |
| Sao Tome and Principe | 134 (97-176) | 211.81 (156.34-274.79) | 272 (196-361) | 261.51 (189.33-341.62) | 0.6  (0.44-0.75) | 1.03 (0.56-1.63) |
| Saudi Arabia | 18628 (13098-25197) | 324.27 (236.09-439.53) | 60772 (44191-81137) | 349.64 (261.69-456.77) | 0.44  (0.19-0.69) | 2.26 (1.25-3.53) |
| Senegal | 6894 (4857-9327) | 217.91 (155.56-295.21) | 14175 (9959-19681) | 193.03 (138.6-264.76) | -0.32  (-0.48--0.17) | 1.06 (0.52-1.73) |
| Serbia | 8254 (5935-10828) | 72.68 (53.49-94.46) | 12770 (9042-17151) | 78.32 (56.45-103.68) | -0.16  (-0.32-0) | 0.55 (0.16-0.96) |
| Seychelles | 149 (122-177) | 264.21 (215.62-313.75) | 412 (335-492) | 372.98 (305.03-442.58) | 0.73  (0.38-1.08) | 1.76 (1.4-2.21) |
| Sierra Leone | 3006 (2108-3920) | 159.22 (113.57-207.13) | 5466 (3882-7506) | 152.78 (111.06-206.6) | 0.06  (-0.04-0.16) | 0.82 (0.35-1.41) |
| Singapore | 3318 (2792-3825) | 150.4 (128.31-172.54) | 7652 (6381-8986) | 98.49 (82.28-115.58) | -0.09  (-0.51-0.33) | 1.31 (1.05-1.58) |
| Slovakia | 4636 (3423-5828) | 77.34 (57.93-96.09) | 4943 (3643-6561) | 53.58 (40.18-70.47) | -0.32  (-0.75-0.12) | 0.07 (-0.14-0.32) |
| Slovenia | 730 (564-937) | 29.99 (23.11-38.33) | 1153 (861-1546) | 26.58 (19.7-35.54) | -0.23  (-0.4--0.06) | 0.58 (0.18-1.02) |
| Solomon Islands | 679 (457-953) | 395.87 (273.36-541.96) | 991 (700-1323) | 252.19 (187.96-323.89) | -1.76  (-2.13--1.38) | 0.46 (0.05-1) |
| Somalia | 4439 (3044-6247) | 180.61 (127.7-249.75) | 10942 (7577-15274) | 171.59 (120.84-235.94) | -0.04  (-0.08-0) | 1.47 (0.87-2.31) |
| South Africa | 21771 (16449-27696) | 101.51 (77.65-127.19) | 67448 (51749-83850) | 154.13 (119.37-188.18) | 2.13  (1.81-2.45) | 2.1 (1.73-2.52) |
| South Sudan | 3411 (2309-4887) | 148.71 (101.01-212.15) | 5206 (3433-7392) | 146.15 (98.14-201.98) | -0.07  (-0.1--0.04) | 0.53 (0.12-1.06) |
| Spain | 27204 (20247-34916) | 49.37 (37.36-62.9) | 33541 (25024-44643) | 30.68 (23.28-40.45) | -1.68  (-1.81--1.55) | 0.23 (0.03-0.46) |
| Sri Lanka | 23983 (19649-28947) | 213.19 (175.66-255.14) | 56366 (42319-75015) | 221.34 (166.69-292.99) | 0.28  (-0.03-0.58) | 1.35 (0.79-2.16) |
| Sudan | 15493 (10740-22010) | 167.34 (115.87-236.78) | 32036 (21410-47847) | 178.04 (120.44-262.15) | -0.07  (-0.29-0.16) | 1.07 (0.41-1.95) |
| Suriname | 492 (387-598) | 185.13 (147.04-223.53) | 1823 (1402-2284) | 299.3 (231.49-372.41) | 1.66  (1.4-1.93) | 2.71 (2.09-3.44) |
| Sweden | 3725 (2978-4582) | 23.69 (18.91-28.99) | 7665 (6086-9355) | 33 (26.21-40.06) | 1.23  (1.02-1.43) | 1.06 (0.84-1.31) |
| Switzerland | 3344 (2620-4186) | 31.22 (24.5-38.97) | 7296 (5588-9367) | 37.38 (29.22-47.43) | 0.9  (0.78-1.02) | 1.18 (0.9-1.52) |
| Syrian Arab Republic | 11780 (8793-15199) | 223.42 (166.98-291.97) | 20562 (14789-27887) | 171.97 (126.92-227.45) | -1.49  (-1.77--1.2) | 0.75 (0.27-1.41) |
| Taiwan (Province of China) | 31802 (26546-36560) | 208.12 (177.61-238.78) | 76647 (60535-95461) | 192.9 (151.81-239.45) | -0.25  (-0.38--0.12) | 1.41 (1.02-1.91) |
| Tajikistan | 828 (657-1019) | 27.44 (21.77-33.62) | 3468 (2618-4402) | 65.82 (50.48-82.7) | 3.38  (3.03-3.72) | 3.19 (2.49-4.12) |
| Thailand | 94341 (74648-115587) | 247.68 (200.65-294.93) | 241434 (179082-316908) | 237.01 (176.44-311.43) | 0.01  (-0.13-0.14) | 1.56 (0.88-2.47) |
| Timor-Leste | 891 (613-1251) | 277.43 (201.41-389.03) | 2274 (1631-3023) | 274.4 (199.2-358.71) | -0.07  (-0.29-0.15) | 1.55 (0.83-2.43) |
| Togo | 2181 (1552-2905) | 177.16 (128.04-233.88) | 6039 (4243-8321) | 166.77 (122.01-223.72) | -0.2  (-0.25--0.15) | 1.77 (1.05-2.64) |
| Tokelau | 4 (3-5) | 286.59 (212.57-388.94) | 5 (3-6) | 343.82 (255.94-470.76) | 0.6  (0.4-0.8) | 0.22 (-0.09-0.6) |
| Tonga | 144 (109-196) | 251.24 (194.65-337.9) | 284 (216-383) | 355.13 (269.87-477.49) | 1.15  (0.83-1.48) | 0.97 (0.53-1.58) |
| Trinidad and Tobago | 1173 (936-1409) | 139.04 (111.21-167.2) | 4228 (3039-5670) | 225.11 (162.65-303.6) | 1.96  (1.71-2.2) | 2.6 (1.76-3.61) |
| Tunisia | 6547 (4909-8429) | 133.79 (101.8-172.1) | 17396 (12579-23467) | 140.75 (101.83-189.11) | 0.23  (0.15-0.31) | 1.66 (0.92-2.57) |
| Turkey | 82136 (62027-112712) | 234.68 (176.43-324.6) | 138083 (107163-174808) | 159.32 (124.36-201.07) | -0.74  (-0.96--0.52) | 0.68 (0.18-1.2) |
| Turkmenistan | 2255 (1724-2777) | 106.16 (81.64-130.33) | 6465 (4669-8574) | 148.58 (109.24-194.47) | 0.65  (0.34-0.96) | 1.87 (1.29-2.61) |
| Tuvalu | 24 (18-32) | 337.05 (251.91-448.27) | 43 (30-59) | 409.76 (293.83-565.31) | 0.65  (0.47-0.84) | 0.77 (0.31-1.39) |
| Uganda | 8709 (6192-11841) | 141.11 (100.58-192.57) | 19075 (13657-25940) | 144.21 (105.48-193.75) | -0.17  (-0.31--0.04) | 1.19 (0.67-1.83) |
| Ukraine | 7555 (5719-9816) | 10.99 (8.31-14.1) | 11226 (8411-14540) | 15.26 (11.48-19.7) | 1.37  (1.19-1.56) | 0.49 (0.31-0.7) |
| United Arab Emirates | 1757 (1231-2352) | 412.04 (272.38-528.87) | 14789 (9048-23941) | 337.67 (215.62-525.05) | -0.65  (-1.15--0.15) | 7.42 (4.59-11.42) |
| United Kingdom | 22310 (17108-28459) | 24.41 (18.8-30.81) | 29495 (22907-37612) | 22.93 (17.68-29.29) | 0.02  (-0.18-0.22) | 0.32 (0.23-0.42) |
| United Republic of Tanzania | 12647 (9097-17072) | 120.74 (88.23-160.93) | 29038 (22554-36513) | 126.84 (99.46-157.47) | 0.17  (0.1-0.23) | 1.3 (0.72-1.96) |
| United States of America | 173949 (135311-214805) | 55.23 (42.86-68.25) | 677949 (543302-807843) | 122.51 (98.96-145.8) | 2.91  (2.55-3.28) | 2.9 (2.53-3.31) |
| United States Virgin Islands | 126 (98-159) | 144.36 (114.07-179.35) | 373 (290-458) | 204.58 (160.66-252.25) | 1.7  (1.48-1.91) | 1.96 (1.36-2.63) |
| Uruguay | 2719 (2416-3041) | 68.96 (61.29-76.9) | 4953 (4166-5815) | 90.09 (76.15-105.04) | 1.35  (1.12-1.58) | 0.82 (0.57-1.09) |
| Uzbekistan | 14449 (10578-20562) | 119.05 (86.31-169.65) | 37161 (28077-47367) | 157.38 (121.21-196.84) | 0.57  (-0.18-1.31) | 1.57 (0.85-2.24) |
| Vanuatu | 153 (104-218) | 216.56 (150.23-304.65) | 659 (456-914) | 361.28 (255.66-495.04) | 1.87  (1.74-1.99) | 3.31 (2.08-5.15) |
| Venezuela (Bolivarian Republic of) | 12453 (10022-14868) | 125.88 (102.03-149.2) | 79062 (56931-105496) | 267.84 (194.88-354.99) | 2.19  (1.72-2.66) | 5.35 (3.85-7.17) |
| Viet Nam | 93396 (70632-120241) | 229.91 (174.92-296.81) | 191203 (142410-251491) | 208.42 (158.71-269.88) | -0.37  (-0.81-0.07) | 1.05 (0.43-1.69) |
| Yemen | 7994 (5411-11724) | 163.57 (113.17-239.37) | 20433 (14406-27821) | 157.22 (113.51-211.36) | -0.18  (-0.28--0.09) | 1.56 (0.92-2.46) |
| Zambia | 4911 (3521-6368) | 177.18 (128.79-227.48) | 11500 (8296-15502) | 178.05 (131.64-238.22) | -0.27  (-0.49--0.05) | 1.34 (0.72-2.1) |
| Zimbabwe | 4352 (2902-6711) | 109.71 (74.24-168.41) | 12362 (8067-19261) | 179.97 (119.98-279.91) | 1.95  (1.59-2.31) | 1.84 (1.2-2.65) |
