# Supplementary material for: Epidemiological patterns of chronic kidney disease attributed to type 2 diabetes from 1990-2019
Source: Front Endocrinol (Lausanne). 2024 Apr 17;15:1383777. doi: 10.3389/fendo.2024.1383777 (PMC11061475; doi:10.3389/fendo.2024.1383777)
Supplement: Supplementary file 7 [file Table_3.docx]

**Supplementary Table 3.** Age distribution of DALY rate for Chronic kidney disease attributed to type 2 diabetes mellitus in different countries in 2019.

| location | <20 years | 20-54 years | 55-59 years | 60-79 years | 80+ years |
| --- | --- | --- | --- | --- | --- |
| Afghanistan | 0.539 | 113.452 | 1014.554 | 1841.047 | 2449.429 |
| Albania | 0.139 | 16.328 | 79.276 | 178.205 | 233.768 |
| Algeria | 0.317 | 56.277 | 393.32 | 888.307 | 2113.418 |
| American Samoa | 4.031 | 274.932 | 1446.534 | 2116.429 | 3238.316 |
| Andorra | 0.026 | 8.922 | 50.665 | 163.78 | 604.435 |
| Angola | 0.5 | 36.037 | 316.516 | 620.503 | 1164.143 |
| Antigua and Barbuda | 0.853 | 105.991 | 671.276 | 1219.752 | 2183.105 |
| Argentina | 0.166 | 45.385 | 355.88 | 859.27 | 1749.944 |
| Armenia | 0.275 | 37.736 | 202.236 | 408.826 | 752.735 |
| Australia | 0.023 | 6.638 | 41.92 | 115.065 | 357.219 |
| Austria | 0.036 | 10.804 | 64.494 | 259.854 | 1118.282 |
| Azerbaijan | 1.06 | 67.936 | 351.625 | 578.703 | 958.289 |
| Bahamas | 0.898 | 116.011 | 587.387 | 954.956 | 1337.863 |
| Bahrain | 0.348 | 41.873 | 362.529 | 960.598 | 2667.998 |
| Bangladesh | 0.153 | 23.471 | 232.891 | 356.898 | 521.457 |
| Barbados | 0.802 | 92.602 | 463.528 | 860.303 | 1367.922 |
| Belarus | 0.047 | 8.109 | 40.135 | 66.591 | 126.063 |
| Belgium | 0.029 | 8.931 | 50.85 | 163.272 | 621.696 |
| Belize | 1.327 | 123.788 | 896.917 | 1277.319 | 1701.395 |
| Benin | 0.537 | 52.641 | 427.964 | 855.054 | 1640.84 |
| Bermuda | 0.294 | 58.834 | 269.911 | 492.112 | 901.948 |
| Bhutan | 0.406 | 55.33 | 531.902 | 941.59 | 1255.831 |
| Bolivia (Plurinational State of) | 0.495 | 75.939 | 749.621 | 1699.687 | 2931.085 |
| Bosnia and Herzegovina | 0.088 | 21.285 | 136.715 | 328.41 | 514.774 |
| Botswana | 0.408 | 69.851 | 550.43 | 943.112 | 1688.376 |
| Brazil | 0.314 | 42.138 | 269.063 | 511.625 | 910.281 |
| Brunei Darussalam | 0.742 | 84.754 | 614.137 | 1449.146 | 3338.756 |
| Bulgaria | 0.167 | 47.77 | 250.106 | 403.414 | 427.439 |
| Burkina Faso | 0.518 | 47.067 | 409.618 | 810.916 | 1588.408 |
| Burundi | 0.47 | 31.902 | 326.079 | 685.813 | 1189.605 |
| Cabo Verde | 0.444 | 41.927 | 314.548 | 614.823 | 1469.063 |
| Cambodia | 2.62 | 99.537 | 546.63 | 768.801 | 1082.076 |
| Cameroon | 1.106 | 83.277 | 629.433 | 1211.494 | 2434.926 |
| Canada | 0.026 | 12.31 | 71.912 | 210.383 | 541.004 |
| Central African Republic | 0.897 | 59.821 | 543.269 | 842.213 | 1172.283 |
| Chad | 0.494 | 50.594 | 434.388 | 870.448 | 1417.326 |
| Chile | 0.098 | 28.77 | 216.783 | 596.081 | 1341.043 |
| China | 0.552 | 45.883 | 187.814 | 384.31 | 779.866 |
| Colombia | 0.272 | 42.072 | 286.615 | 606.631 | 995.137 |
| Comoros | 0.496 | 39.509 | 321.204 | 767.664 | 1417.322 |
| Congo | 0.592 | 54.932 | 437.42 | 816.084 | 1592.912 |
| Cook Islands | 1.344 | 161.523 | 745.67 | 1130.827 | 1922.002 |
| Costa Rica | 0.348 | 72.304 | 510.44 | 945.579 | 1419.675 |
| Côte d'Ivoire | 0.573 | 56.546 | 437.636 | 867.34 | 1655.631 |
| Croatia | 0.059 | 18.234 | 105.452 | 293.876 | 567.61 |
| Cuba | 0.312 | 61.701 | 283.067 | 524.57 | 621.922 |
| Cyprus | 0.025 | 9.223 | 78.109 | 320.523 | 1382.3 |
| Czechia | 0.046 | 15.061 | 85.065 | 206.491 | 362.432 |
| Democratic People's Republic of Korea | 1.217 | 91.178 | 402.587 | 620.841 | 720.261 |
| Democratic Republic of the Congo | 0.551 | 37.097 | 345.257 | 641.805 | 1155.92 |
| Denmark | 0.028 | 9.193 | 58.73 | 200.784 | 577.671 |
| Djibouti | 0.515 | 39.686 | 351.652 | 791.382 | 1559.796 |
| Dominica | 1.692 | 153.048 | 814.052 | 1460.268 | 2355.534 |
| Dominican Republic | 0.507 | 74.686 | 455.502 | 720.332 | 1013.217 |
| Ecuador | 0.594 | 78.226 | 703.346 | 1369.882 | 2701.065 |
| Egypt | 0.316 | 69.297 | 740.018 | 1657.718 | 2502.017 |
| El Salvador | 1.101 | 218.775 | 1561.008 | 2306.024 | 2426.24 |
| Equatorial Guinea | 0.816 | 35.028 | 378.898 | 832.138 | 1953.986 |
| Eritrea | 0.614 | 42.752 | 418.64 | 781.328 | 1342.57 |
| Estonia | 0.056 | 11.557 | 72.641 | 217.328 | 586.94 |
| Eswatini | 0.746 | 105.549 | 849.199 | 1403.424 | 2366.523 |
| Ethiopia | 0.779 | 28.302 | 284.966 | 743.452 | 1560.089 |
| Fiji | 4.163 | 200.982 | 1124.029 | 1742.18 | 2875.112 |
| Finland | 0.025 | 6.776 | 36.079 | 119.184 | 453.062 |
| France | 0.023 | 6.595 | 40.149 | 121.155 | 439.103 |
| Gabon | 1 | 63.962 | 523.013 | 1066.229 | 2320.549 |
| Gambia | 0.542 | 47.343 | 438.704 | 874.933 | 1612.256 |
| Georgia | 0.4 | 68.638 | 285.152 | 460.974 | 500.803 |
| Germany | 0.035 | 12.11 | 74.97 | 288.021 | 1218.002 |
| Ghana | 0.692 | 61.689 | 450.736 | 882.343 | 1850.848 |
| Greece | 0.036 | 15.67 | 99.438 | 332.125 | 902.988 |
| Greenland | 0.026 | 19.266 | 121.851 | 301.127 | 625.514 |
| Grenada | 1.383 | 151.443 | 874.826 | 1670.551 | 2107.746 |
| Guam | 2.466 | 184.275 | 853.386 | 1101.236 | 1538.549 |
| Guatemala | 1.537 | 123.533 | 1161.74 | 1835.38 | 2759.382 |
| Guinea | 0.568 | 57.034 | 474.522 | 902.573 | 1524.695 |
| Guinea-Bissau | 0.845 | 80.923 | 680.341 | 1129.658 | 1809.259 |
| Guyana | 1.249 | 162.578 | 1131.288 | 1542.853 | 1791.291 |
| Haiti | 0.713 | 89.367 | 692.072 | 1091.231 | 1315.466 |
| Honduras | 0.514 | 108.488 | 1211.103 | 2038.259 | 2383.61 |
| Hungary | 0.071 | 17.844 | 107.187 | 247.151 | 544.174 |
| Iceland | 0.018 | 4.879 | 31.321 | 88.758 | 274.283 |
| India | 0.343 | 58.132 | 479.495 | 710.069 | 784.422 |
| Indonesia | 4.795 | 121.952 | 535.404 | 769.055 | 994.74 |
| Iran (Islamic Republic of) | 0.427 | 36.631 | 271.431 | 622.122 | 1376.117 |
| Iraq | 0.525 | 76.547 | 740.547 | 1539.875 | 2572.439 |
| Ireland | 0.029 | 8.057 | 51.169 | 167.209 | 601.907 |
| Israel | 0.039 | 15.808 | 151.279 | 496.136 | 1612.467 |
| Italy | 0.034 | 8.335 | 44.804 | 145.395 | 545.833 |
| Jamaica | 0.712 | 112.736 | 677.278 | 1004.46 | 1337.196 |
| Japan | 0.103 | 27.224 | 141.674 | 366.006 | 976.203 |
| Jordan | 0.381 | 58.814 | 538.132 | 1389.538 | 2419.719 |
| Kazakhstan | 0.267 | 50.77 | 261.985 | 445.694 | 536.695 |
| Kenya | 0.588 | 30.808 | 277.82 | 615.405 | 1332.096 |
| Kiribati | 4.978 | 307.081 | 1611.431 | 1989.581 | 2604.793 |
| Kuwait | 0.184 | 26.85 | 224.04 | 600.54 | 1181.682 |
| Kyrgyzstan | 0.478 | 56.449 | 279.325 | 309.364 | 254.716 |
| Lao People's Democratic Republic | 4.274 | 174.829 | 1162.793 | 1753.037 | 2256.818 |
| Latvia | 0.057 | 10.224 | 52.753 | 120.619 | 205.885 |
| Lebanon | 0.242 | 42.387 | 341.239 | 861.607 | 1432.646 |
| Lesotho | 0.614 | 96.611 | 811.585 | 1294.919 | 2041.173 |
| Liberia | 0.599 | 58.692 | 437.353 | 846.335 | 1731.775 |
| Libya | 0.396 | 68.243 | 506.566 | 1159.196 | 1610.565 |
| Lithuania | 0.054 | 8.611 | 43.949 | 96.331 | 145.204 |
| Luxembourg | 0.03 | 9.264 | 59.053 | 195.395 | 697.734 |
| Madagascar | 0.414 | 29.957 | 275.561 | 551.162 | 983.646 |
| Malawi | 0.64 | 31.198 | 327.996 | 748.356 | 1361.113 |
| Malaysia | 1.488 | 87.055 | 643.403 | 1196.798 | 1626.579 |
| Maldives | 1.3 | 72.835 | 596.904 | 1233.963 | 2667.221 |
| Mali | 0.507 | 51.695 | 422 | 815.978 | 1491.546 |
| Malta | 0.038 | 10.24 | 72.303 | 240.11 | 618.428 |
| Marshall Islands | 3.366 | 267.971 | 1480.263 | 1803.365 | 2550.03 |
| Mauritania | 0.455 | 48.35 | 387.347 | 807.147 | 1761.602 |
| Mauritius | 8.382 | 437.47 | 2250.773 | 3560.301 | 3826.388 |
| Mexico | 1.621 | 180.914 | 1318.452 | 2060.407 | 2518.122 |
| Micronesia (Federated States of) | 5.762 | 390.904 | 2221.203 | 2659.529 | 3723.049 |
| Monaco | 0.027 | 9.24 | 45.309 | 147.361 | 442.826 |
| Mongolia | 0.378 | 84.63 | 341.873 | 496.572 | 647.868 |
| Montenegro | 0.116 | 30.538 | 196.543 | 420.709 | 615.797 |
| Morocco | 0.37 | 66.187 | 553.367 | 1201.527 | 1952.84 |
| Mozambique | 0.652 | 35.75 | 351.175 | 675.055 | 1376.582 |
| Myanmar | 5.42 | 160.861 | 644.315 | 953.339 | 1541.036 |
| Namibia | 0.217 | 39.121 | 335.278 | 657.655 | 1188.445 |
| Nauru | 4.639 | 306.998 | 1783.432 | 2165.971 | 3459.504 |
| Nepal | 0.379 | 57.517 | 503.353 | 848.355 | 990.658 |
| Netherlands | 0.024 | 7.059 | 42.355 | 145.803 | 583.087 |
| New Zealand | 0.044 | 14.22 | 80.694 | 172.273 | 394.028 |
| Nicaragua | 0.799 | 178.708 | 1697.873 | 2927.349 | 3362.796 |
| Niger | 0.411 | 42.976 | 377.537 | 741.592 | 1349.222 |
| Nigeria | 0.394 | 31.984 | 248.217 | 561.938 | 1307.088 |
| Niue | 4.069 | 256.654 | 1235.374 | 1806.86 | 2621.048 |
| North Macedonia | 0.101 | 29.546 | 170.994 | 377.297 | 471.781 |
| Northern Mariana Islands | 3.892 | 328.379 | 1327.593 | 1951.114 | 3255.955 |
| Norway | 0.027 | 5.675 | 33.508 | 115.89 | 475.891 |
| Oman | 0.174 | 17.008 | 214.99 | 734.48 | 1745.357 |
| Pakistan | 1.129 | 100.053 | 742.819 | 1110.053 | 1339.446 |
| Palau | 4.682 | 462.514 | 1694.01 | 2400.396 | 3534.356 |
| Palestine | 0.383 | 51.707 | 528.754 | 1266.65 | 1974.011 |
| Panama | 0.523 | 79.676 | 514.724 | 942.877 | 1472.494 |
| Papua New Guinea | 1.838 | 83.843 | 432.283 | 526.846 | 634.496 |
| Paraguay | 0.451 | 70.653 | 672.003 | 1236.049 | 1554.096 |
| Peru | 0.383 | 42.578 | 310.883 | 686.237 | 1349.893 |
| Philippines | 4.393 | 215.167 | 1183.73 | 1688.139 | 2048.432 |
| Poland | 0.079 | 14.669 | 89.612 | 199.733 | 371.273 |
| Portugal | 0.039 | 12.11 | 72.105 | 243.388 | 888.997 |
| Puerto Rico | 0.365 | 98.892 | 531.344 | 974.503 | 1579.645 |
| Qatar | 0.208 | 21.346 | 263.776 | 912.865 | 4437.787 |
| Republic of Korea | 0.104 | 27.791 | 156.307 | 416.579 | 1072.976 |
| Republic of Moldova | 0.07 | 9.146 | 55.554 | 96.149 | 141.244 |
| Romania | 0.071 | 19.998 | 108.405 | 226.851 | 248.491 |
| Russian Federation | 0.084 | 11.439 | 64.97 | 119.346 | 172.136 |
| Rwanda | 0.491 | 30.379 | 301.668 | 658.762 | 1369.809 |
| Saint Kitts and Nevis | 1.145 | 155.519 | 1030.711 | 1744.23 | 2424.454 |
| Saint Lucia | 0.9 | 130.939 | 639.883 | 1041.523 | 1677.925 |
| Saint Vincent and the Grenadines | 1.187 | 149.667 | 682.302 | 1081.93 | 1836.259 |
| Samoa | 2.821 | 208.096 | 1176.804 | 1620.333 | 2385.893 |
| San Marino | 0.025 | 6.812 | 37.004 | 117.098 | 377.352 |
| Sao Tome and Principe | 0.809 | 86.002 | 662.639 | 1227.378 | 2378.316 |
| Saudi Arabia | 0.415 | 92.568 | 867.722 | 1748.408 | 2808.666 |
| Senegal | 0.65 | 57.402 | 463.657 | 927.392 | 1845.364 |
| Serbia | 0.049 | 23.734 | 165.913 | 439.042 | 829.246 |
| Seychelles | 2.292 | 198.736 | 948.228 | 1723.339 | 2984.584 |
| Sierra Leone | 0.532 | 46.386 | 377.332 | 739.892 | 1375.843 |
| Singapore | 0.156 | 28.454 | 213.664 | 519.605 | 1028.086 |
| Slovakia | 0.07 | 20.465 | 127.731 | 294.099 | 434.993 |
| Slovenia | 0.036 | 11.352 | 54.654 | 134.978 | 307.703 |
| Solomon Islands | 2.895 | 205.547 | 868.696 | 837.952 | 877.485 |
| Somalia | 0.648 | 41.52 | 470.55 | 854.489 | 1362.944 |
| South Africa | 0.309 | 55.137 | 374.659 | 702.996 | 1606.616 |
| South Sudan | 0.484 | 35.921 | 316.313 | 759.324 | 1410.269 |
| Spain | 0.019 | 6.605 | 38.766 | 142.614 | 633.805 |
| Sri Lanka | 2.122 | 116.429 | 540.474 | 1055.395 | 1686.328 |
| Sudan | 0.283 | 42.941 | 431.974 | 964.456 | 1430.07 |
| Suriname | 1.408 | 153.107 | 915.858 | 1453.348 | 1975.815 |
| Sweden | 0.019 | 6.329 | 41.9 | 162.528 | 674.813 |
| Switzerland | 0.03 | 7.64 | 45.678 | 168.54 | 816.376 |
| Syrian Arab Republic | 0.674 | 63.927 | 394.708 | 873.114 | 1608.666 |
| Taiwan (Province of China) | 0.546 | 77.182 | 436.763 | 940.936 | 2090.11 |
| Tajikistan | 0.312 | 32.878 | 142.026 | 259.734 | 561.648 |
| Thailand | 1.897 | 135.295 | 573.706 | 1141.791 | 1812.693 |
| Timor-Leste | 2.416 | 106.755 | 826.952 | 1330.177 | 1750.389 |
| Togo | 0.521 | 56.815 | 425.25 | 788.308 | 1476.402 |
| Tokelau | 2.127 | 210.153 | 1111.444 | 1449.443 | 2218.385 |
| Tonga | 2.48 | 184.189 | 1129.196 | 1612.924 | 2290.401 |
| Trinidad and Tobago | 0.683 | 115.312 | 710.134 | 1112.468 | 1346.72 |
| Tunisia | 0.195 | 41.953 | 306.532 | 745.346 | 1361.567 |
| Turkey | 0.299 | 42.61 | 329.081 | 861.023 | 1578.884 |
| Turkmenistan | 1.128 | 107.069 | 459.607 | 635.933 | 441.849 |
| Tuvalu | 3.451 | 244.061 | 1296.941 | 1698.396 | 2495.935 |
| Uganda | 0.532 | 29.583 | 316.968 | 746.249 | 1431.645 |
| Ukraine | 0.072 | 9.432 | 41.323 | 64.179 | 135.61 |
| United Arab Emirates | 0.561 | 116.349 | 812.634 | 1534.8 | 2849.578 |
| United Kingdom | 0.033 | 7.854 | 40.644 | 111.241 | 347.53 |
| United Republic of Tanzania | 0.246 | 25.96 | 272.726 | 666.852 | 1321.819 |
| United States of America | 0.05 | 48.389 | 278.489 | 635.302 | 1202.466 |
| United States Virgin Islands | 0.466 | 122.044 | 583.161 | 1018.921 | 1520.464 |
| Uruguay | 0.115 | 27.912 | 176.291 | 481.378 | 1040.819 |
| Uzbekistan | 1.165 | 93.424 | 416.38 | 670.409 | 887.4 |
| Vanuatu | 2.515 | 194.244 | 1178.188 | 1509.158 | 2051.505 |
| Venezuela (Bolivarian Republic of) | 0.571 | 107.966 | 800.469 | 1430.653 | 1618.301 |
| Viet Nam | 2.615 | 96.371 | 478.153 | 929.657 | 1913.935 |
| Yemen | 0.2 | 38.777 | 406.15 | 834.295 | 1188.048 |
| Zambia | 0.754 | 44.627 | 435.38 | 896.731 | 1621.113 |
| Zimbabwe | 0.334 | 58.08 | 483.351 | 849.334 | 1485.691 |
