# Supplementary material for: Epidemiological patterns of chronic kidney disease attributed to type 2 diabetes from 1990-2019
Source: Front Endocrinol (Lausanne). 2024 Apr 17;15:1383777. doi: 10.3389/fendo.2024.1383777 (PMC11061475; doi:10.3389/fendo.2024.1383777)
Supplement: Supplementary file 8 [file Table_4.docx]

**Supplementary Table 4** Age distribution of Incidence rate for Chronic kidney disease attributed to type 2 diabetes mellitus in different countries in 2019.

| location | <20 years | 20-54 years | 55-59 years | 60-79 years | 80+ years |
| --- | --- | --- | --- | --- | --- |
| Afghanistan | 0.035 | 12.476 | 127.247 | 330.812 | 251.223 |
| Albania | 0.07 | 8.663 | 60.471 | 163.489 | 120.174 |
| Algeria | 0.027 | 16.025 | 154.872 | 406.214 | 227.628 |
| American Samoa | 0.093 | 18.935 | 115.53 | 223.9 | 144.763 |
| Andorra | 0.013 | 5.487 | 40.229 | 202.536 | 266.743 |
| Angola | 0.027 | 3.425 | 39.344 | 94.137 | 89.025 |
| Antigua and Barbuda | 0.077 | 19.822 | 129.505 | 238.712 | 144.916 |
| Argentina | 0.017 | 7.147 | 63.078 | 241.935 | 287.888 |
| Armenia | 0.09 | 9.437 | 66.918 | 124.064 | 75.146 |
| Australia | 0.015 | 5.15 | 50.466 | 281.332 | 280.181 |
| Austria | 0.013 | 6.275 | 51.746 | 265.179 | 283.458 |
| Azerbaijan | 0.123 | 11.095 | 77.378 | 127.27 | 75.477 |
| Bahamas | 0.072 | 17.304 | 108.264 | 193.343 | 129.267 |
| Bahrain | 0.034 | 19.984 | 200.186 | 473.615 | 180.426 |
| Bangladesh | 0.03 | 6.163 | 52.922 | 104.155 | 96.453 |
| Barbados | 0.065 | 18.753 | 105.94 | 207.347 | 143.256 |
| Belarus | 0.052 | 9.551 | 45.557 | 79.588 | 78.448 |
| Belgium | 0.013 | 5.729 | 45.617 | 226.058 | 296.539 |
| Belize | 0.076 | 15.278 | 117.235 | 195.326 | 127.526 |
| Benin | 0.049 | 4.666 | 55.206 | 143.681 | 100.479 |
| Bermuda | 0.045 | 19.68 | 109.932 | 229.12 | 143.462 |
| Bhutan | 0.067 | 8.366 | 69.396 | 142.517 | 105.523 |
| Bolivia (Plurinational State of) | 0.029 | 8.765 | 79.937 | 206.388 | 209.68 |
| Bosnia and Herzegovina | 0.052 | 11.271 | 75.305 | 187.391 | 123.119 |
| Botswana | 0.042 | 8.073 | 78.878 | 183.987 | 144.592 |
| Brazil | 0.028 | 11.812 | 81.188 | 185.848 | 159.928 |
| Brunei Darussalam | 0.037 | 9.818 | 92.807 | 266.918 | 339.971 |
| Bulgaria | 0.067 | 14.175 | 79.989 | 183.535 | 116.218 |
| Burkina Faso | 0.038 | 4.31 | 50.687 | 129.026 | 98.226 |
| Burundi | 0.02 | 2.605 | 34.918 | 86.151 | 82.363 |
| Cabo Verde | 0.056 | 5.407 | 54.655 | 140.617 | 96.431 |
| Cambodia | 0.034 | 7.457 | 63.579 | 123.059 | 108.321 |
| Cameroon | 0.079 | 6.623 | 84.265 | 193.345 | 106.098 |
| Canada | 0.014 | 5.077 | 44.165 | 200.051 | 278.454 |
| Central African Republic | 0.037 | 3.762 | 36.013 | 77.759 | 79.367 |
| Chad | 0.041 | 4.104 | 49.187 | 126.469 | 92.839 |
| Chile | 0.021 | 8.78 | 77.981 | 292.815 | 284.389 |
| China | 0.02 | 7.086 | 39.176 | 128.154 | 170.751 |
| Colombia | 0.042 | 15.335 | 123.968 | 238.921 | 173.932 |
| Comoros | 0.037 | 3.644 | 39.43 | 99.493 | 90.18 |
| Congo | 0.037 | 4.638 | 45.219 | 105.548 | 95.604 |
| Cook Islands | 0.066 | 18.159 | 102.533 | 210.948 | 139.564 |
| Costa Rica | 0.063 | 25.544 | 303.037 | 305.205 | 168.009 |
| Côte d'Ivoire | 0.062 | 5.125 | 60.243 | 151.524 | 104.472 |
| Croatia | 0.04 | 11.035 | 77.291 | 236.271 | 132.552 |
| Cuba | 0.049 | 18.504 | 98.755 | 181.189 | 119.769 |
| Cyprus | 0.013 | 5.21 | 55.565 | 285.429 | 335.108 |
| Czechia | 0.034 | 10.242 | 67.169 | 194.152 | 124.992 |
| Democratic People's Republic of Korea | 0.042 | 9.122 | 52.312 | 139.888 | 147.642 |
| Democratic Republic of the Congo | 0.028 | 3.297 | 38.337 | 90.35 | 89.999 |
| Denmark | 0.013 | 6.052 | 46.835 | 228.541 | 265.191 |
| Djibouti | 0.032 | 3.695 | 42.048 | 104.193 | 93.661 |
| Dominica | 0.088 | 18.821 | 115.232 | 221.397 | 153.266 |
| Dominican Republic | 0.048 | 12.089 | 85.712 | 155.962 | 123.534 |
| Ecuador | 0.053 | 13.8 | 117.184 | 272.243 | 231.503 |
| Egypt | 0.029 | 16.441 | 177.773 | 415.826 | 212.095 |
| El Salvador | 0.104 | 24.118 | 162.278 | 248.375 | 157.681 |
| Equatorial Guinea | 0.038 | 3.306 | 48.314 | 122.644 | 104.93 |
| Eritrea | 0.038 | 3.275 | 39.173 | 85.356 | 84.204 |
| Estonia | 0.059 | 12.87 | 74.062 | 141.931 | 87.879 |
| Eswatini | 0.055 | 8.489 | 80.031 | 175.88 | 144.206 |
| Ethiopia | 0.02 | 2.418 | 34.17 | 92.447 | 86.966 |
| Fiji | 0.056 | 14.059 | 96.565 | 186.483 | 140.238 |
| Finland | 0.012 | 4.049 | 29.791 | 169.815 | 243.315 |
| France | 0.014 | 5.052 | 40.609 | 199.962 | 275.621 |
| Gabon | 0.049 | 5.482 | 56.189 | 139.044 | 113.886 |
| Gambia | 0.055 | 4.395 | 54.169 | 142.012 | 99.662 |
| Georgia | 0.097 | 11.921 | 65.956 | 108.95 | 60.104 |
| Germany | 0.016 | 7.386 | 60.699 | 258.209 | 294.288 |
| Ghana | 0.057 | 4.781 | 51.175 | 133.161 | 102.041 |
| Greece | 0.014 | 7.347 | 54.37 | 263.317 | 272.357 |
| Greenland | 0.013 | 6.048 | 51.308 | 179.55 | 286.02 |
| Grenada | 0.085 | 20.026 | 125.818 | 258.59 | 139.35 |
| Guam | 0.058 | 16.164 | 86.923 | 170.573 | 120.587 |
| Guatemala | 0.069 | 16.294 | 162.525 | 271.077 | 211.142 |
| Guinea | 0.052 | 4.525 | 52.393 | 136.749 | 95.708 |
| Guinea-Bissau | 0.07 | 4.739 | 55.008 | 130.662 | 95.368 |
| Guyana | 0.067 | 17.233 | 115.929 | 185.158 | 131.939 |
| Haiti | 0.041 | 9.86 | 84.042 | 149.402 | 129.161 |
| Honduras | 0.054 | 15.899 | 147.196 | 220.99 | 165.726 |
| Hungary | 0.041 | 10.652 | 67.437 | 192.357 | 124.744 |
| Iceland | 0.011 | 4.227 | 37.176 | 165.675 | 255.709 |
| India | 0.046 | 7.233 | 58.436 | 146.72 | 113.142 |
| Indonesia | 0.05 | 8.966 | 58.949 | 117.537 | 109.914 |
| Iran (Islamic Republic of) | 0.026 | 17.744 | 142.095 | 335.074 | 211.542 |
| Iraq | 0.041 | 16.602 | 184.823 | 421.159 | 182.024 |
| Ireland | 0.019 | 7.255 | 55.609 | 233.404 | 219.54 |
| Israel | 0.013 | 7.003 | 68.144 | 288.488 | 277.003 |
| Italy | 0.014 | 5.36 | 37.95 | 204.929 | 288.758 |
| Jamaica | 0.062 | 15.489 | 102.216 | 186.089 | 120.895 |
| Japan | 0.024 | 12.197 | 78.052 | 279.545 | 287.322 |
| Jordan | 0.036 | 17.964 | 190.135 | 450.689 | 183.286 |
| Kazakhstan | 0.101 | 11.083 | 70.734 | 109.623 | 66.095 |
| Kenya | 0.028 | 2.945 | 34.22 | 88.614 | 88.24 |
| Kiribati | 0.086 | 11.079 | 71.697 | 121.29 | 109.046 |
| Kuwait | 0.03 | 15.992 | 168.401 | 399.301 | 174.474 |
| Kyrgyzstan | 0.137 | 8.492 | 53.255 | 74.777 | 51.054 |
| Lao People's Democratic Republic | 0.052 | 10.042 | 88.698 | 162.973 | 121.419 |
| Latvia | 0.057 | 11.664 | 58.967 | 104.986 | 78.72 |
| Lebanon | 0.026 | 17.176 | 176.269 | 458.268 | 172.836 |
| Lesotho | 0.045 | 7.366 | 65.979 | 145.165 | 129.82 |
| Liberia | 0.054 | 4.89 | 53.416 | 139.467 | 97.795 |
| Libya | 0.045 | 17.615 | 162.116 | 396.528 | 191.571 |
| Lithuania | 0.057 | 10.463 | 51.668 | 91.757 | 73.014 |
| Luxembourg | 0.013 | 5.852 | 47.833 | 242.757 | 290.178 |
| Madagascar | 0.027 | 2.831 | 33.638 | 77.329 | 75.637 |
| Malawi | 0.031 | 2.764 | 39.04 | 98.123 | 92.359 |
| Malaysia | 0.041 | 11.213 | 107.758 | 209.449 | 122.012 |
| Maldives | 0.029 | 9.694 | 120.28 | 248.916 | 131.92 |
| Mali | 0.034 | 5.594 | 53.748 | 117.64 | 98.624 |
| Malta | 0.014 | 5.823 | 53.746 | 257.668 | 265.916 |
| Marshall Islands | 0.074 | 12.677 | 85.647 | 153.892 | 141.32 |
| Mauritania | 0.054 | 5.5 | 61.038 | 158.781 | 98.96 |
| Mauritius | 0.099 | 28.191 | 174.045 | 287.306 | 110.027 |
| Mexico | 0.09 | 30.922 | 221.892 | 319.349 | 159.425 |
| Micronesia (Federated States of) | 0.115 | 17.173 | 108.571 | 185.624 | 134.884 |
| Monaco | 0.013 | 6.786 | 43.584 | 213.993 | 254.013 |
| Mongolia | 0.111 | 10.487 | 68.393 | 107.765 | 69.897 |
| Montenegro | 0.069 | 13.294 | 92.05 | 223.1 | 134.303 |
| Morocco | 0.036 | 16.197 | 153.038 | 380.213 | 205.747 |
| Mozambique | 0.031 | 2.917 | 37.192 | 88.782 | 92.196 |
| Myanmar | 0.071 | 10.948 | 78.241 | 156.326 | 122.977 |
| Namibia | 0.029 | 6.633 | 69.084 | 164.161 | 130.461 |
| Nauru | 0.089 | 12.98 | 96.725 | 161.084 | 162.606 |
| Nepal | 0.072 | 10.522 | 79.641 | 145.577 | 99.506 |
| Netherlands | 0.011 | 5.06 | 39.165 | 220.178 | 301.368 |
| New Zealand | 0.023 | 7.209 | 56.1 | 260.858 | 263.716 |
| Nicaragua | 0.092 | 29.573 | 197.47 | 249.094 | 150.618 |
| Niger | 0.037 | 3.831 | 47.386 | 121.47 | 94.551 |
| Nigeria | 0.034 | 4.443 | 48.36 | 138.18 | 106.309 |
| Niue | 0.081 | 20.29 | 110.838 | 207.103 | 129.966 |
| North Macedonia | 0.054 | 12.648 | 88.776 | 226.014 | 135.271 |
| Northern Mariana Islands | 0.119 | 27.251 | 123.283 | 222.97 | 136.182 |
| Norway | 0.012 | 3.942 | 33.377 | 179.025 | 269.499 |
| Oman | 0.024 | 9.141 | 158.453 | 415.478 | 200.566 |
| Pakistan | 0.063 | 8.084 | 62.955 | 131.04 | 114.47 |
| Palau | 0.109 | 24.264 | 118.435 | 213.54 | 134.073 |
| Palestine | 0.031 | 14.045 | 179.962 | 428.29 | 180.42 |
| Panama | 0.055 | 19.933 | 146.334 | 261.24 | 169.517 |
| Papua New Guinea | 0.033 | 5.835 | 40.264 | 74.79 | 80.908 |
| Paraguay | 0.035 | 13.98 | 120.821 | 220.401 | 147.139 |
| Peru | 0.031 | 9.737 | 84.646 | 218.479 | 191.251 |
| Philippines | 0.06 | 12.914 | 101.504 | 187.544 | 131.218 |
| Poland | 0.027 | 8.285 | 57.15 | 172.053 | 150.854 |
| Portugal | 0.012 | 6.233 | 45.381 | 215.545 | 276.745 |
| Puerto Rico | 0.053 | 22.08 | 131.117 | 255.017 | 132.517 |
| Qatar | 0.027 | 12.622 | 203.746 | 491.378 | 205.266 |
| Republic of Korea | 0.018 | 7.675 | 55.956 | 198.337 | 305.475 |
| Republic of Moldova | 0.055 | 8.31 | 45.545 | 67.876 | 63.471 |
| Romania | 0.044 | 10.999 | 66.386 | 168.388 | 116.172 |
| Russian Federation | 0.06 | 14.587 | 69.371 | 104.405 | 77.633 |
| Rwanda | 0.024 | 2.89 | 39.084 | 94.799 | 91.774 |
| Saint Kitts and Nevis | 0.062 | 25.973 | 165.69 | 255.616 | 146.978 |
| Saint Lucia | 0.077 | 19.328 | 115.837 | 216.753 | 154.024 |
| Saint Vincent and the Grenadines | 0.063 | 17.41 | 97.189 | 190.652 | 148.975 |
| Samoa | 0.075 | 14.129 | 98.508 | 183.867 | 134.163 |
| San Marino | 0.013 | 4.824 | 35.841 | 183.115 | 257.877 |
| Sao Tome and Principe | 0.094 | 7.219 | 71.371 | 181.169 | 109.164 |
| Saudi Arabia | 0.06 | 21.677 | 226.196 | 496.014 | 132.233 |
| Senegal | 0.05 | 4.606 | 51.814 | 135.329 | 100.411 |
| Serbia | 0.037 | 10.973 | 80.381 | 216.309 | 132.589 |
| Seychelles | 0.049 | 17.501 | 125.238 | 258.09 | 137.93 |
| Sierra Leone | 0.053 | 3.854 | 47.467 | 129.3 | 95.159 |
| Singapore | 0.02 | 10.274 | 82.366 | 247.259 | 264.18 |
| Slovakia | 0.033 | 10.828 | 76.916 | 189.274 | 112.454 |
| Slovenia | 0.035 | 8.932 | 55.296 | 192.401 | 142.061 |
| Solomon Islands | 0.092 | 9.725 | 57.653 | 101.543 | 117.749 |
| Somalia | 0.029 | 2.595 | 35.834 | 81.87 | 84.163 |
| South Africa | 0.038 | 8.59 | 79.189 | 185.335 | 147.419 |
| South Sudan | 0.025 | 3.308 | 35.86 | 91.253 | 84.536 |
| Spain | 0.008 | 5.158 | 39.439 | 209.857 | 302.696 |
| Sri Lanka | 0.046 | 14.557 | 106.078 | 240.95 | 128.136 |
| Sudan | 0.024 | 10.476 | 126.588 | 329.4 | 230.81 |
| Suriname | 0.051 | 20.469 | 129.501 | 202.832 | 135.569 |
| Sweden | 0.007 | 3.403 | 31.225 | 170.618 | 204.07 |
| Switzerland | 0.014 | 5.362 | 42.647 | 234.674 | 296.128 |
| Syrian Arab Republic | 0.051 | 20.064 | 153.781 | 384.92 | 227.625 |
| Taiwan (Province of China) | 0.041 | 14.686 | 100.325 | 263.998 | 188.228 |
| Tajikistan | 0.095 | 6.218 | 47.693 | 84.775 | 65.11 |
| Thailand | 0.06 | 16.862 | 111.766 | 256.716 | 129.796 |
| Timor-Leste | 0.038 | 8.173 | 78.305 | 150.692 | 116.246 |
| Togo | 0.056 | 4.963 | 54.286 | 136.298 | 99.75 |
| Tokelau | 0.056 | 14.111 | 88.903 | 177.929 | 139.375 |
| Tonga | 0.055 | 13.649 | 94.125 | 181.586 | 127.705 |
| Trinidad and Tobago | 0.056 | 18.944 | 120.268 | 201.419 | 129.29 |
| Tunisia | 0.029 | 17.092 | 157.476 | 420.166 | 193.118 |
| Turkey | 0.031 | 14.926 | 144.014 | 400.219 | 195.247 |
| Turkmenistan | 0.17 | 10.905 | 68.089 | 96.208 | 54.187 |
| Tuvalu | 0.07 | 12.758 | 86.565 | 163.524 | 133.736 |
| Uganda | 0.019 | 2.319 | 33.414 | 87.503 | 88.098 |
| Ukraine | 0.053 | 8.04 | 36.189 | 70.716 | 81.057 |
| United Arab Emirates | 0.038 | 21.562 | 222.499 | 453.826 | 198.532 |
| United Kingdom | 0.017 | 5.141 | 37.949 | 201.902 | 273.72 |
| United Republic of Tanzania | 0.018 | 3.076 | 39.442 | 103.788 | 96.409 |
| United States of America | 0.025 | 10.254 | 84.646 | 279.024 | 279.803 |
| United States Virgin Islands | 0.06 | 21.343 | 113.737 | 220.565 | 143.625 |
| Uruguay | 0.017 | 5.567 | 48.596 | 198.321 | 263.354 |
| Uzbekistan | 0.178 | 11.225 | 85.976 | 132.699 | 70.323 |
| Vanuatu | 0.051 | 9.537 | 72.212 | 137.267 | 132.248 |
| Venezuela (Bolivarian Republic of) | 0.057 | 23.469 | 164.499 | 266.524 | 156.884 |
| Viet Nam | 0.039 | 8.25 | 67.766 | 155.158 | 127.466 |
| Yemen | 0.021 | 8.594 | 111.82 | 293.474 | 235.744 |
| Zambia | 0.037 | 3.345 | 45.379 | 111.074 | 98.037 |
| Zimbabwe | 0.031 | 6.218 | 62.873 | 143.883 | 131.054 |
